# Phosphonation of Alginate–Polyethyleneimine Beads for the Enhanced Removal of Cs(I) and Sr(II) from Aqueous Solutions

**DOI:** 10.3390/gels9020152

**Published:** 2023-02-11

**Authors:** Khalid A. M. Salih, Kanggen Zhou, Mohammed F. Hamza, Hamed Mira, Yuezhou Wei, Shunyan Ning, Eric Guibal, Waheed M. Salem

**Affiliations:** 1 School of Metallurgy and Environment, Central South University, Changsha 410083, China; 2 School of Nuclear Science and Technology, University of South China, Hengyang 421001, China; 3 Nuclear Materials Authority, POB 530, El-Maadi, Cairo 11728, Egypt; 4 School of Nuclear Science and Engineering, Shanghai Jiao Tong University, Shanghai 200240, China; 5 Polymers Composites and Hybrids (PCH), IMT Mines Ales, CEDEX, F-30319 Alès, France; 6 Medical Labs Department, Faculty of Applied Health Science Technology, Menoufia University, Shebine El-Koam 6131567, Egypt

**Keywords:** metal sorption, composite hydrogel, functionalization, sorption isotherm, uptake kinetics, selectivity, modeling, reuse cycles, cesium, strontium

## Abstract

Although Cs(I) and Sr(II) are not strategic and hazardous metal ions, their recovery from aqueous solutions is of great concern for the nuclear industry. The objective of this work consists of designing a new sorbent for the simultaneous recovery of these metals with selectivity against other metals. The strategy is based on the functionalization of algal/polyethyleneimine hydrogel beads by phosphonation. The materials are characterized by textural, thermo-degradation, FTIR, elemental, titration, and SEM-EDX analyses to confirm the chemical modification. To evaluate the validity of this modification, the sorption of Cs(I) and Sr(II) is compared with pristine support under different operating conditions: the pH effect, kinetics, and isotherms are investigated in mono-component and binary solutions, before investigating the selectivity (against competitor metals) and the possibility to reuse the sorbent. The functionalized sorbent shows a preference for Sr(II), enhanced sorption capacities, a higher stability at recycling, and greater selectivity against alkali, alkaline-earth, and heavy metal ions. Finally, the sorption properties are compared for Cs(I) and Sr(II) removal in a complex solution (seawater sample). The combination of these results confirms the superiority of phosphonated sorbent over pristine support with promising performances to be further evaluated with effluents containing radionuclides.

## 1. Introduction

Cesium is mainly used in many applications, including drilling fluids, optical glasses, catalyst promoters, the manufacture of vacuum tubes and solar cells and panels, and radiation monitoring [1]. Relatively abundant and commercially exploited as a by-product from the extraction of valuable metals (such as lithium), this natural metal is not considered a hazardous element, since it is easily excreted [2]. The main concern for cesium element regards its radioactive form (as ^137^Cs) [3,4], occurring in nuclear effluents [5,6]. The Fukushima Daiichi accident attracted the attention of the community due to its dispersion in the marine environment [7]. Recently, the attention on cesium was also driven by the necessity to develop strategies for the treatment and valorization of spent solar cells and panels [8]. The case of strontium differs somewhat. Due its capacity to be assimilated by the organism, strontium is more hazardous: it may replace calcium in bones and cause rachitic lesions, and affect intestinal, renal, and kidney functions [9]. However, like cesium, strontium is especially critical when issued from nuclear reactions (as ^90^Sr) [10,11]. This is also a relatively common resource; however, for geostrategic reasons (concentrated production in a limited number of countries), the evaluation of its supply risk is relatively high [1]. This metal is used for fireworks, flares, ferrite magnets, glow-in-the-dark paintings, and special machinery applications [1].

The treatment of effluents containing these metal ions may involve different processes, depending on the relative concentrations, the flow rate, and the composition: precipitation [12] (including micro-precipitation assisted by reagents such as Prussian blue [13], combined co-precipitation into porous sorbents [14]), solvent extraction [15], and adsorption techniques [16,17]. Solvent extraction was reported for the treatment of these metal ions, mainly for nuclear effluents (Hanford tanks) [18]. The removal of cesium from aqueous solutions by sorption has been widely investigated with different strategies involving ion-exchange sorbents [6], composite materials [19], nanostructured inorganic solids [20,21], and biosorbents [22]. Many studies have reported the interest for Prussian blue (and analogs, based on hexacyanoferrates) for the sequestration of cesium, based on the remarkable steric arrangement of its crystalline structure that provides outstanding selectivity for this metal, even in complex solutions and at low concentration [23,24,25,26,27]. The affinity of Cs(I) for Prussian blue is driven by the radius of Cs(I) that is shorter than the crystal cage size, while other compounds cannot accommodate this sieving effect [25]. Therefore, the simultaneous binding of other hazardous metals requires synthesizing dual-functional sorbents [28,29]. Alginate (extracted from brown algal biomass, ALG), bearing mannuronic and guluronic acid groups, possesses attractive properties for the binding of metal cations (through the complexation of divalent and trivalent cations by carboxylic groups [30]). This interaction is also used for the ionotropic gelation of the biopolymer, which, in turn, can be used for incorporating an ion-exchanger, sorbents, microparticles [31]. Based on this conclusion, the design of a sorbent elaborated fromalginate-like support with simultaneous Cs(I) and Sr(II) binding properties, but with relative selectivity against alkali, alkaline-earth, and heavy metals, is of great interest and the strategy may involve manufacturing materials with different functional groups, with complementary reactivity. Hydrogels are remarkable sorbents because of their attractive diffusion properties (decrease in the crystallinity of biopolymers such as chitosan, expansion of the polymer network, etc.). Polyethyleneimine-based hydrogels have also attracted significant attention because of the density of amine groups that can bind metal anion species (in acidic solutions, when amine groups are protonated) and metal cations (through complexation in near-neutral solutions) [32,33]. This interest is also reinforced by the ability of amine groups to be post-functionalized. The synthesis of composite materials associating different polymers is attracting increasing attention so that the bi-functionality of sorbents can be taken advantage of [34,35,36].

Previous studies in our group have shown the ready fabrication of a highly reactive support resulting from interactions between alginate (and/or algal biomass, submitted to partial alginate extraction) and polyethyleneimine (branched PEI, herein named PEI). Combing the interactions between the carboxylic groups of alginate and the amine groups of PEI, with complementary cross-linking (glutaraldehyde with amine groups of PEI) and ionotropic gelation of alginate leads to the production of stable spherical hydrogels that can bind metal ions directly [37]. However, these pristine beads are also characterized by their ready ability to be chemically modified by grafting new functional groups to provide dual-binding sites: amidoxime [38,39], quaternary ammonium [40], and sulfonate [41]. These beads have also been phosphorylated for the recovery of Nd(III) and Mo(VI) [42]. The efficiency of phosphonate-based sorbents for recovering cesium and strontium has already been documented [43,44]. Hereafter, a new process of phosphorylation of pristine algal/PEI beads (ALG-PEI) is proposed for producing APO-PEI beads and applied to the recovery of both Cs(I) and Sr(II). The main objective of this work is to answer the question of whether the grafting of phosphonate moieties brings substantial improvement in sorption properties. To address this, materials (both ALG-PEI and APO-PEI) are extensively characterized before being tested for the sorption of Cs(I) and Sr(II) (mono-component and binary solutions) through the study of pH effect, uptake kinetics, sorption isotherms, selectivity (in the presence of multi-component solutions), and metal desorption (including sorbent recycling). Finally, the sorption process is applied to the removal of Cs(I) and Sr(II) from a complex solution (seawater).

## 2. Results and Discussion

### 2.1. Characterization of Sorbents

#### 2.1.1. Physical Characteristics

**Figure A1:**
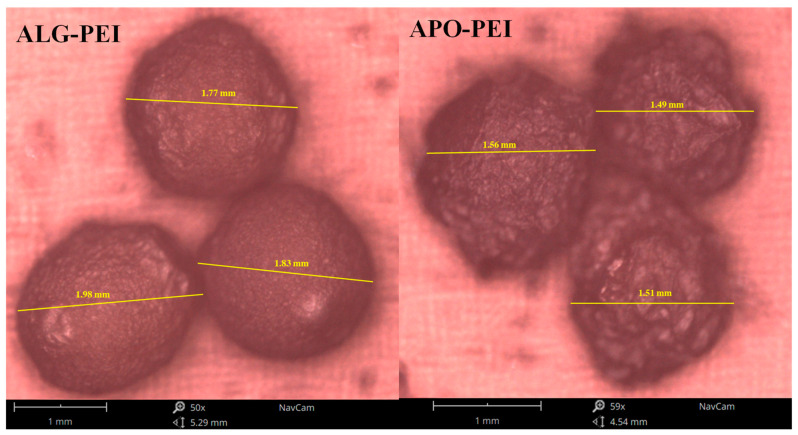
SEM photos for shape and size evaluation of sorbent particles.

Figure A1 shows the roughly spherical morphology of the sorbent beads. The final step in the synthesis consists of air-drying: the shrinking that occurs under uncontrolled drying conditions may explain the irregular surface. The largest irregularities of the surfaces of APO-PEI beads may be correlated with the greater number of drying steps involved in the synthesis (in addition to the fact that ALG-PEI was freeze-dried). The freeze-drying of ALG-PEI in the synthesis procedure allows the shrinking of the material to be minimized (due to the better management of capillary forces in the network of the hydrogel during the drying phase). In the case of APO-PEI, during chemical modification, two supplementary steps of drying were included in the protocol. In these cases, the materials were systematically air-dried. These drying conditions cause a series of shrinking steps, which, in turn, may explain the local collapse of the network provoking more irregularities at the surface of the beads. It is noteworthy that the phosphorylation step leads to a small decrease in the size of the beads: from 1.86 ± 0.09 mm for ALG-PEI to 1.52 ± 0.03 mm for APO-PEI beads.

**Figure A2:**
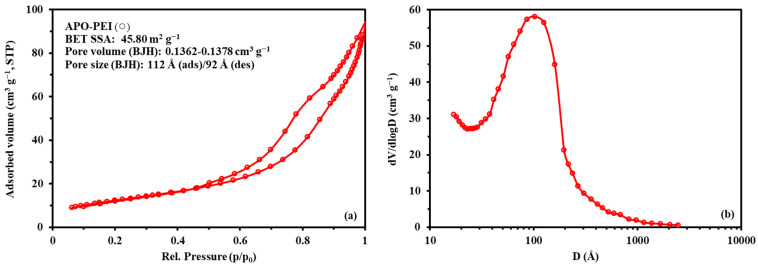
Textural analysis of APO-PEI sorbents: (**a**) N_2_ sorption and desorption isotherms (BET method) and (**b**) pore size distribution (BJH method).

The textural properties of ALG-PEI were previously determined by Hamza et al. [41]: the specific surface area (S_BET_) is close to 6 m^2^ g^−1^, while the porous volume is 0.0224 cm^3^ g^−1^ and the average pore width varies between 155 Å (desorption branch) and 166 Å (adsorption branch). After functionalization, the specific surface increases to 45.8 m^2^ g^−1^, despite the shrinking of the beads (as shown by size variation) (Figure A2a). This is confirmed by the expanded micropore volume (i.e., 0.137 cm^3^ g^−1^), while the distribution of pore size decreases (i.e., 112 Å/92 Å, for adsorption and desorption, respectively). The shape of the sorption isotherms corresponds to the Type IIb profile according to the classification of Rouquerol [45], while the hysteresis loop can be assimilated to Type B [46]. Type II isotherms are associated with non-porous or macro-porous structures, while Type B loop hysteresis corresponds to a slit-type geometry of pores [47]. These characteristics are consistent with the profiles observed for ALG-PEI sorbent [41] (apart from the expansion of the micro-pore volume and the increase in S_BET_). This is also consistent with the variations of the textural parameters observed after sulfonation of pristine beads [41].

**Figure A3:**
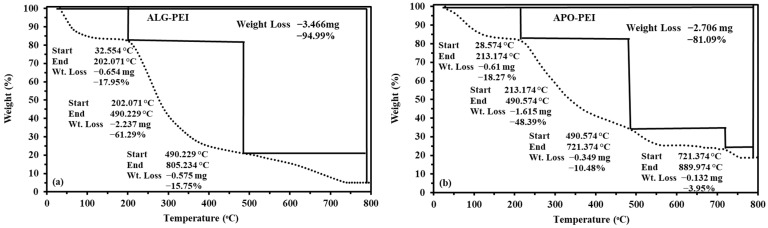

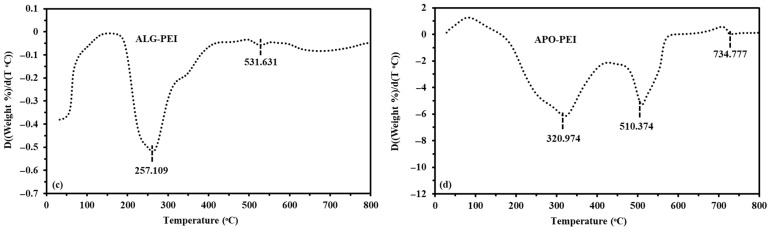
Characterization of thermal degradation of ALG-PEI (**a**,**c**) and APO-PEI sorbents (**b**,**d**): TGA curves (weight loss, (**a**,**b**)) and DTG curves (**c**,**d**).

Figure A3 compares the TGA (weight loss) and DTG curves for the two sorbents. The phosphorylation of ALG-PEI (to form APO-PEI) increases the thermal stability of the sorbent. This is demonstrated by both the residual weight at 800 °C, which increases from 5% to 18.9%, and the existence of a fourth degradation step (in the higher temperature range). The differences in the profiles are clearly identified in the DTG curves (Figure A3b), where a single extremum is observed for ALG-PEI at 257.1 °C, while for APO-PEI, two extrema can be detected at higher temperatures: 321.0 °C (shift of ALG-PEI extremum) and 510.4 °C (new extremum). This improvement in thermal stability is directly correlated to the presence of phosphonate groups. Indeed, the phosphonation of supports is a well-known method for improving their fire-retardant properties [48].

**Figure A4:**
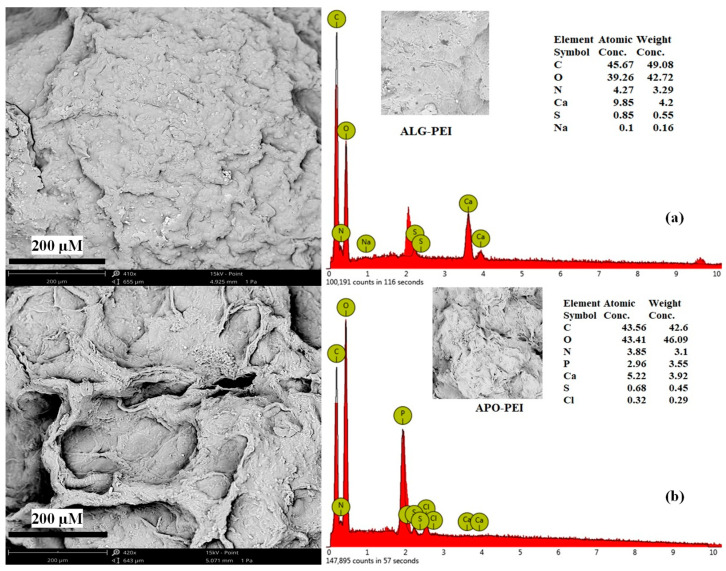
SEM observation (**left** panels) and semi-quantitative EDX analysis (**right** panels) of ALG-PEI (**a**) and APO-PEI (**b**).

Figure A4 shows the scanning electron microscopy (SEM) images of the surface of the ALG-PEI bead (Figure A4a) and APO-PEI beads (Figure A4b). The figure clearly shows that the functionalization of pristine beads alters the surface of the material with the formation of fractures and pores, and more irregularities (which support the higher specific surface area of APO-PEI).

#### 2.1.2. Chemical Characteristics

**Figure A5:**
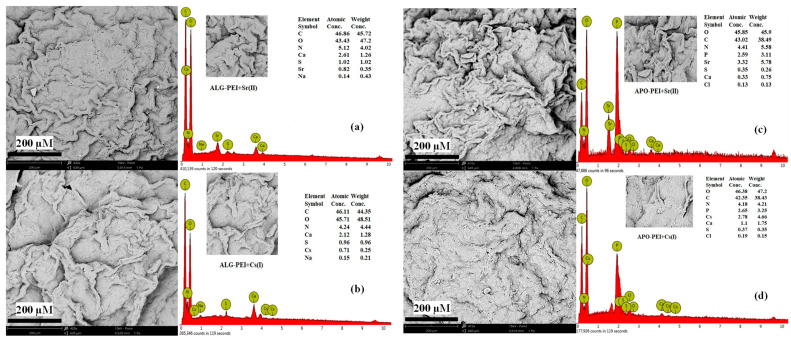
SEM observation (**left** panels) and semi-quantitative EDX analysis (**right** panels) of ALG-PEI after Sr(II) sorption (**a**) and Cs(I) sorption (**b**), and of APO-PEI after Sr(II) sorption (**c**) and Cs(I) sorption (**d**).

**Figure A6:**
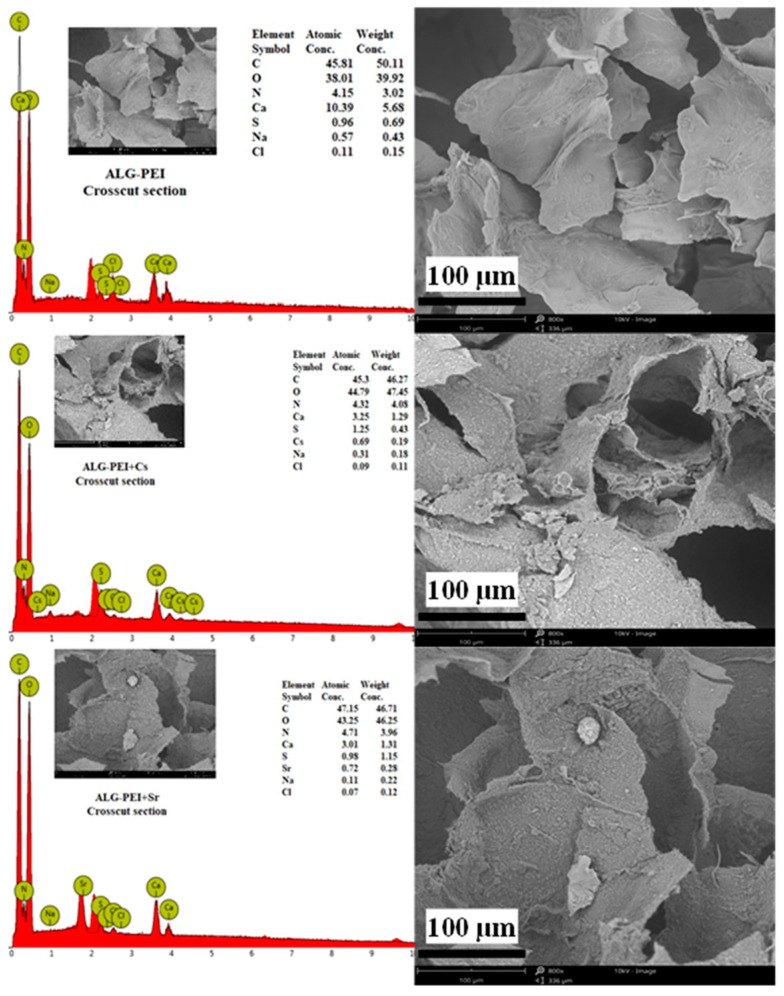
SEM-EDX analysis of cross-sections of ALG-PEI sorbent before and after Cs(I) and Sr(II) sorption.

**Figure A7:**
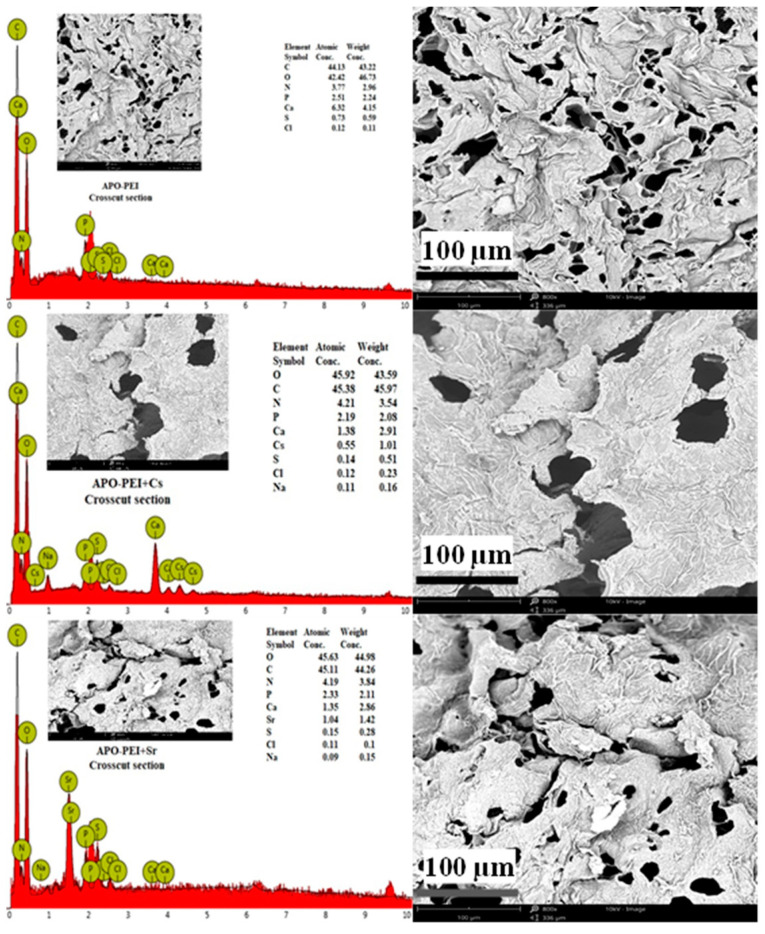
SEM-EDX analysis of cross-sections of APO-PEI sorbent before and after Cs(I) and Sr(II) sorption.

In Figure A4, the semi-quantitative EDX analysis of the surface of the sorbents confirms the efficient grafting of the phosphonate-based compound: the O and P contents significantly increase after chemical modification. It is noteworthy that Ca content decreases after functionalization. The presence of Cl elements (although of low content) means that the epichlorohydrin linker is not fully saturated during the synthesis. Figure A5 shows the SEM observations and the semi-quantitative EDX analysis of the two sorbents after Sr(II) and Cs(I) sorption. It is noteworthy that regardless of the sorbed metal, the Ca content strongly decreases at the surface of ALG-PEI (from 9.85 At.% to 2.6–2.1 At.%); this is most likely associated with the ion-exchange of Ca(II) with Cs(I) and Sr(II). On the other hand, for APO-PEI, calcium content decreases from 5.5 At.% to 1.1 At.% for Cs(I) and down to 0.3 At.% for Sr(II). For APO-PEI, the sorption of metal ions involves a contribution of Ca(II) ion-exchange (which is more important in the case of Sr(II)). The comparison of the semi-quantitative analysis of the cross-sections of ALG-PEI (Figure A6) and APO-PEI (Figure A7) shows that the composition is roughly homogeneous at the surface and in the center of the beads. In the case of ALG-PEI, the concentration levels of Cs(I) and Sr(II) are comparable between the surface of the sorbent and at the surface of the internal scaffolds. In contrast, for APO-PEI, when the semi-quantitative EDX analyses of Cs(I) and Sr(II) reach atomic concentrations close to 2.78% and 3.32% at the surface, respectively, the contents drop to 0.55% and 1.04% within the bead, respectively. Despite the good textural properties of APO-PEI and the homogeneous distribution of reactive groups (reflected by the comparison of S, P, N, and O semi-quantitative contents between external and internal surfaces), a concentration difference can be identified.

**Scheme 1:**
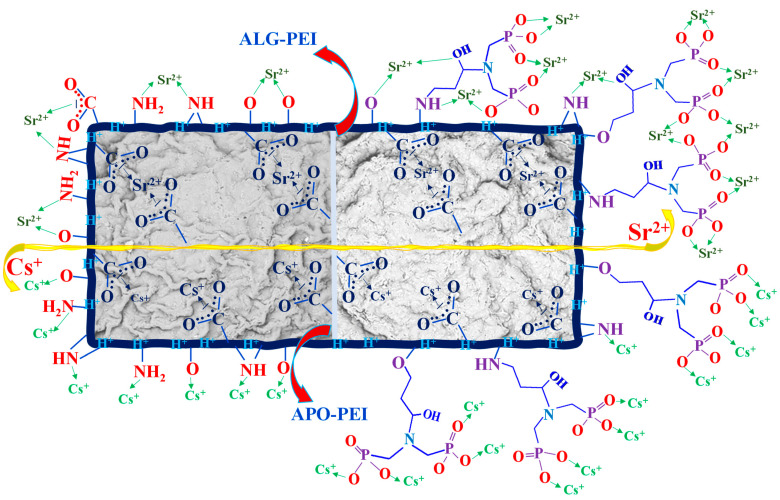
Prospective binding mechanisms for Cs(I) and Sr(II) sorption onto ALG-PEI and APO-PEI sorbents.

**Figure 1:**
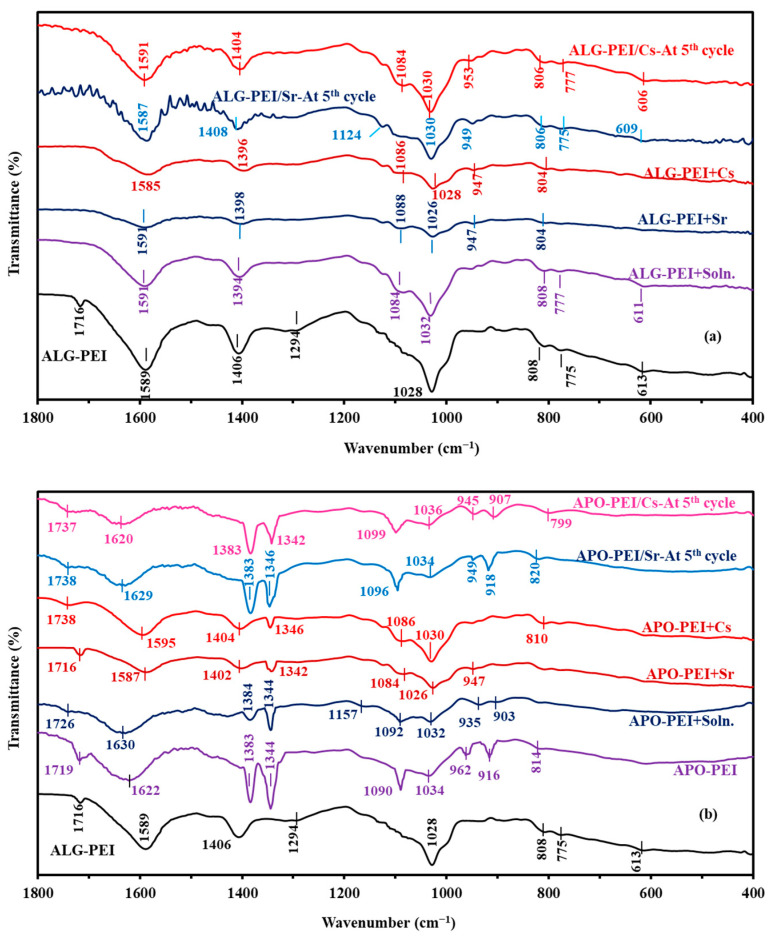
FTIR spectra of ALG-PEI (**a**) and APO-PEI (**b**) sorbents at different stages of use: pristine material, after conditioning at pH 7 (the pH of metal sorption, referenced as “sorbent + Soln.”), after Cs(I) or Sr(II) sorption, after five cycles of sorption/desorption; wavenumber range: 1800–400 cm^−1^.

Figure 1 compiles the FTIR spectra (1800–400 cm^−1^ wavenumber range) of ALG-PEI beads (Figure 1a) and APO-PEI beads (Figure 1b) at different stages of their use (pristine, pH 7-conditioned sorbents, after metal sorption, and after five cycles of reuse). Pristine (reference) spectra allow the identification of functional groups present on the sorbents. The spectra for the 4000–1800 cm^−1^ wavenumber range are not reported hereafter. A wide and poorly resolved band is observed around 3280 cm^−1^ for ALG-PEI; this band is usually associated with ν_N-H_ and ν_O-H_ (and their convolution). In the case of functionalized material, the band shifts to ≈3428 cm^−1^. This is probably due to the increase in the density of –OH groups (in the phosphonate moieties). Two other bands are identified at 2850–2875 cm^−1^ and 2920–2960 cm^−1^, which are assigned to ν_C-H_ vibrations. They are poorly affected by the chemical modification or by metal sorption and desorption operations. More interesting are the spectra in the region 1800–400 cm^−1^. A band is detected in the range 1715–1738 cm^−1^, which is assigned to carbonyl and carboxyl groups (ν_C(=O)O_, ester stretching vibration) [49] or to imide carbonyl symmetrical stretching [50] and the carbamate carbonyl group [51]. The band at 1589 cm^−1^ in ALG-PEI can be associated with the overlapping of ν_C=N_, δ_N-H_ (in primary and secondary amines); depending on the operating conditions, this band may vary between 1585 and 1591 cm^−1^. After phosphorylation, a strong shift is observed toward a higher wavenumber, up to 1622–1630 cm^−1^. It is noteworthy that after Cs(I) and Sr(II) sorption, the band is shifted back to 1587–1595 cm^−1^ (with a relative decrease in intensity and poor resolution), while after metal desorption, the signal is restored to 1620–1629 cm^−1^. Imine and amine groups are involved in metal binding. Surprisingly, this effect is not detected for ALG-PEI (despite the presence of numerous amine groups). The wide band appearing at 1406 cm^−1^ in ALG-PEI is assigned to the salt form of carboxylic acid; after metal binding, the band is shifted toward a lower wavenumber (i.e., 1396–1398 cm^−1^). Again, after metal desorption, the band returns to 1404–1408 cm^−1^. Carboxylic groups are involved in metal binding, most likely through the ion-exchange of Ca(II) with target metals (see Scheme 1 below for suggestions on the binding mechanisms), especially Sr(II) or their complexation with carboxylate groups (deprotonated at pH 7). In the case of APO-PEI, the band at 1406 cm^−1^ disappears and is replaced with two sharp bands at 1383 and 1344 cm^−1^, which may be assigned to (a) the shift of COO^−^ and to P = O/P-OH groups [52], and (b) in-plane δ_O-H_ (in primary and secondary hydroxyl from alginate) and/or P-OH vibrations, respectively. After metal sorption, the 1344 cm^−1^ band is weakly affected, contrary to the band at 1383 cm^−1^, which is replaced by a broader band centered at 1402–1404 cm^−1^. After metal desorption, the bands are restored to 1383/1346–1342 cm^−1^. The phosphonate group is also involved in metal binding. In the range 1000–1200 cm^−1^, the spectra show a series of peaks and shoulders that correspond to the superposition of ν_C-C_, ν_C-O-C_, ν_C-O_, and ν_C-N_ vibrations. The most significant bands are identified at 1090 cm^−1^ (especially in APO-PEI due to an increased contribution of ν_C-N_ vibration, which decreases with metal binding, but recovers with metal elution) and at 1028–1034 cm^−1^ (which is typical of the carbohydrate ring); the broad band around may be explained by the contribution of other groups, such as phosphonate (ν_PO3_) [43,53].

FTIR characterization clearly demonstrates the following:

- The grafting of phosphonic acid functions;
- The contribution of amine and carboxylic groups in the binding of Cs(I) and Sr(II) in the case of ALG-PEI;
- The additional contribution of phosphonate groups in metal binding for APO-PEI;
- The restoration of chemical structure after metal desorption (even after the fifth reuse).

**Figure A8:**
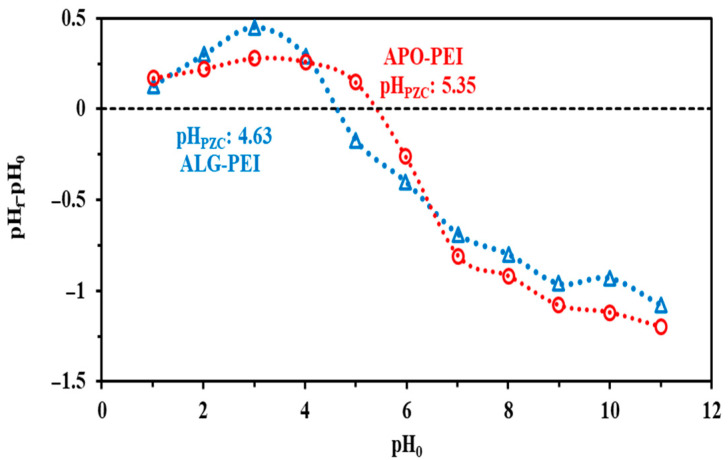
Determination of pH_PZC_ values of ALG-PEI and APO-PEI sorbents (pH-drift method; background salt solution: 0.1 M NaCl; sorbent dose, SD: 2 g L^−1^; time: 48 h; agitation, v: 210 rpm; T: 21 ± 1 °C).

Figure A8 shows that the functionalization slightly increases the pH_PZC_ of ALG-PEI sorbent (from 4.63 to 5.35). The application of the pH-drift method shows very similar titration profiles. The acid–base properties of the sorbent are controlled by the reactive groups present on the sorbent. Alginate (and relevant algal biomass) bears mannuronic and guluronic acid groups (with pK_a_ values close to 3.38 and 3.65, respectively [54]). Phosphonate groups also bring acidic properties: pK_a_ values strongly depend on the substitute groups and chemical environment but, in most cases, the acid groups have values below 3 [55]. On the other hand, branched PEI, bearing primary, secondary, and tertiary amine groups, provides alkaline properties (pK_a_ 4.5, 6.7, and 11.6, respectively). The weakly acid pH_PZC_ values result from the combined effects of these different functionalities. Surprisingly, phosphorylation slightly increases the pH_PZC_ value, meaning that the second acidity of phosphonate (with pK_a_ values in the range 7–9 [55]) modulates the acid–base properties of the grafted functionalization agent. In acidic solutions (below pH 4.63 or 5.35), the surface of the sorbents is positively charged, making the reactive groups less available for the binding of metal cations. APO-PEI sorbent will require slightly higher pH values for optimized sorption conditions.

The elemental analysis of the sorbents is summarized in Table A1. The comparison of molar contents confirms the successful grafting of iminodi(methylphosphonic acid (HN[CH_2_PO(OH)_2_]_2_); indeed, the contents of both nitrogen, oxygen, and phosphorus increase with the chemical modification. However, the increase in molar contents for these elements cannot be directly correlated to their respective contents in the functionalization agent or the synthesis yield. This is probably due to the bulky grafting: the steric hindrance during functionalization affects synthesis yield.

### 2.2. Cs(I) and Sr(II) Sorption from Synthetic Solutions

#### 2.2.1. pH Effect

**Figure 2:**
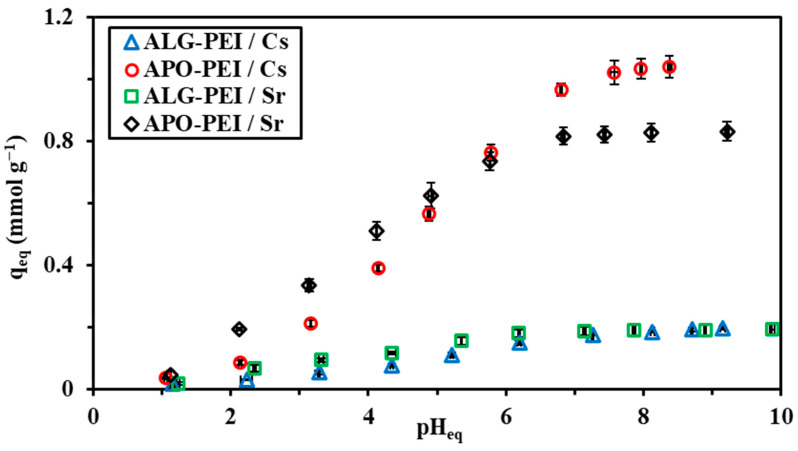
Effect of pH on Cs(I) and Sr(II) sorption using ALG-PEI and APO-PEI sorbents (C_0_: 0.802 mmol Cs L^−1^ or 2.128 mmol Sr L^−1^; sorbent dose, SD: 0.67 g L^−1^; v: 210 rpm; time: 48 h; T: 21 ± 1 °C).

In Figure 2, the comparison of pH-edge curves demonstrate that the functionalization of ALG-PEI beads drastically increases the binding of both Cs(I) and Sr(II). It is noteworthy that for ALG-PEI beads, the profiles are superposed, with limited variations in the sorption capacities (despite initial concentration being 2.5 times larger for Sr(II)). In the case of APO-PEI, the curves almost overlap up to pH_eq_ 5.5, while above this value, the sorbent binds Cs(I) better than Sr(II) (despite the lower molar concentration). Apparently, the sorbents have a greater affinity for Cs(I) than for Sr(II) and the phosphorylation improves this preferential sorption. In acidic solutions, the competition of protons and the protonation of reactive groups almost completely inhibits metal binding. This is consistent with the pH_PZC_ values of the sorbents. With increasing the pH, the competition of the protons decreases; the progressive deprotonation of reactive groups improves the binding of Cs(I) and Sr(II).

**Figure A9:**
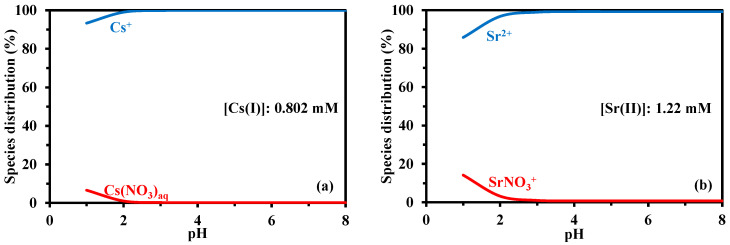
Speciation diagrams of Cs(I) (**a**) and Sr(II) (**b**) (under the experimental conditions selected for the study of pH effect, calculations using Visual MINTEQ software [76]).

The speciation of metal ions may significantly affect their binding onto sorbents due to, for example, the changes in overall charge and ionic size (by the formation of hydrolyzed species). Herein, the speciation of Cs(I) and Sr(II), under the experimental conditions selected for pH study, is revealed to be relatively “monotonous” (Figure A9): above pH 2, free cationic species are the only species present in solution (i.e., Cs^+^ and Sr^2+^); speciation does not affect the sorption of the target metal in the operative pH range.

**Figure A10:**
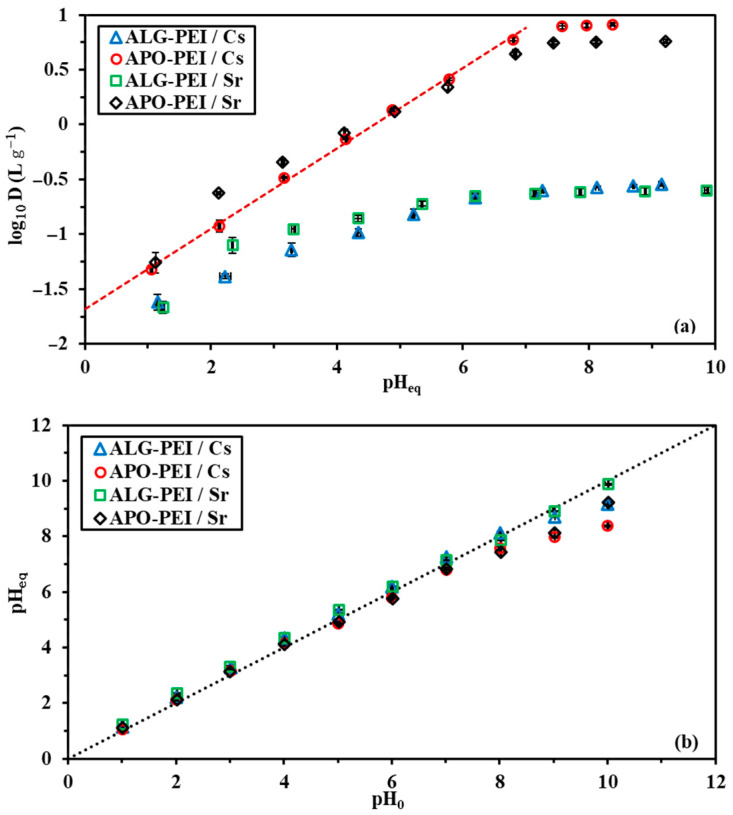
Effect of pH on Cs(I) and Sr(II) sorption: (**a**) log_10_ D vs. pH_eq_ plot and (**b**) pH variation during metal sorption for ALG-PEI and APO-PEI sorbents (C_0_: 0.802 mmol Cs L^−1^ or 2.128 mmol Sr L^−1^; sorbent dose, SD: 0.67 g L^−1^; v: 210 rpm; time: 48 h; T: 21 ± 1 °C).

In Figure A10a, the decimal logarithmic plot of the distribution ratio D (D = q_eq_/C_eq_) vs. equilibrium pH does not show a linear trend, except for Cs(I) sorption using APO-PEI sorbent. The slope of linear fit is used in ion-exchange processes for evaluating the stoichiometry of proton exchange with the target metal and then the stoichiometric ratio between the metal and reactive groups. The co-existence of different reactive groups at the surface of the sorbent may explain the dispersion of data, as well as the unexpected slope analysis for the system Cs(I)/ALG-PEI (i.e., +0.366). The sorption process usually involves parallel pH variations; Figure A10b shows that the pH variation remains negligible (variations < ±0.3 pH unit) below pH 7, while above substantial differences are observed between the different systems. For ALG-PEI/Sr(II), the pH hardly changes, while for the other systems, the pH tends to decrease by up to 0.85 pH units for ALG-PEI/Cs(I), 0.78 pH units for APO-PEI/Sr(II), and up to 1.62 pH units for APO-PEI/Cs(I). The larger pH variation for Cs(I) at high pH values may be explained by differences in hydrolysis phenomena (and the difference in stoichiometric proton exchange between sorbed metal ion and the relevant reactive groups in the sorbent). The higher sensitivity of APO-PEI sorbent in terms of pH variation is correlated with the highest value of pH_PZC_ (Figure A6).

Beaugeard et al. [56] documented the different mechanisms that may be involved in the binding of metal ions through (a) the deprotonation of –OH sites from phosphonic acid groups (for the ion-exchange mechanism), but also (b) the direct complexation of metal cations (via dative O-bonds within R-P(-OH)_2_ groups).

ALG-PEI bears carboxylic acid/carboxylate groups (depending on the pH), as well as amine groups (including primary, secondary, and tertiary amine groups). Both of them may engage ion-exchange interactions and chelation mechanisms depending on the pH [56]. At a pH below the pK_a_ of carboxylic groups of alginate (i.e., 3.4–3.6), metal cations bind to the biosorbent by complexation, while at a pH above the pK_a_, the deprotonation of mannuronic and guluronic acids enables the electrostatic attraction of metal cations. In the case of N-donor functional moieties, these basic reactive groups show a reciprocal trend [56]: at higher pH values, the deprotonation of amine groups allows the binding of cations onto the lone pair of the nitrogen atom. Clearly, the steric hindrance may reduce the reactive pattern of the sorbent. The density of these reactive groups is difficult to be established since some of these sites are engaged in an interpolymer network assembly (alginate/PEI) or glutaraldehyde crosslinking; therefore, both the accessibility and the availability of these reactive groups may be restrained. The phosphonation of ALG-PEI (in APO-PEI) reduces the availability of some of the residual groups but brings additional and complementary phosphonated groups. Beaugeard et al. [56] identified three reaction regimes depending on the pH. Hence, phosphonic acids can be considered diacids; as a corollary, the pK_a_ of reactive groups can be associated with the acid value (i.e., pK_a_ 2–3) and the near-neutral value (i.e., pK_a_ 6–7), respectively. These considerations lead to the conclusion that the binding mechanisms strongly change with pH, as follows:

(a) In the acidic region (pH < 2–3), the reactive groups (not dissociated) bind metal cations through chelation (polychelatogen behavior) at the expense of weak sorption capacities;

(b) In intermediary pH range, the partial deprotonation of reactive groups leads to a mix of mechanisms, such as chelation (polychelatogen behavior onto the non-dissociated site) and ion-exchange (electrostatic interaction of metal cations with dissociated functional groups);

(c) At a higher pH range (neutral region and weakly alkaline), the complete deprotonation of reactive groups leads to electrostatic attraction of metal cations on negatively charged functional groups.

In APO-PEI, the combination of the reactive groups from ALG-PEI (O- and N-donors) and phosphonate compartments offer a wide range of reactive groups and binding mechanisms controlled by the pH according to the rules cited above. It is clear that the size of metal cations (Cs(I) and Sr(II)) may influence their spatial arrangement of the cation in the sorbent, which, in turn, affects the reactivity of the functional groups and the coordination sphere (in the case of the chelation mechanism).

These mechanisms are confirmed not only by the analysis of the effect of the pH and the changes in the FTIR spectra (shift of bands and disappearance of signals), but also by the variation in the composition of the sorbent (for example, the significant decrease in Ca(II) content in the semi-quantitative EDX analyses shows the ion-exchange between metal ions and Ca(II)).

In the process of desorption, the treatment of metal-loaded sorbents with acidic solutions promotes the protonation of reactive groups, which, in turn, facilitates the release of loaded metal ions due to the lower reactivity of functional groups and the competition effect of protons [56]. Scheme 1 shows the suggested mechanisms involved in the binding of Cs(I) and Sr(II) onto both ALG-PEI and APO-PEI sorbents.

**Figure A11:**
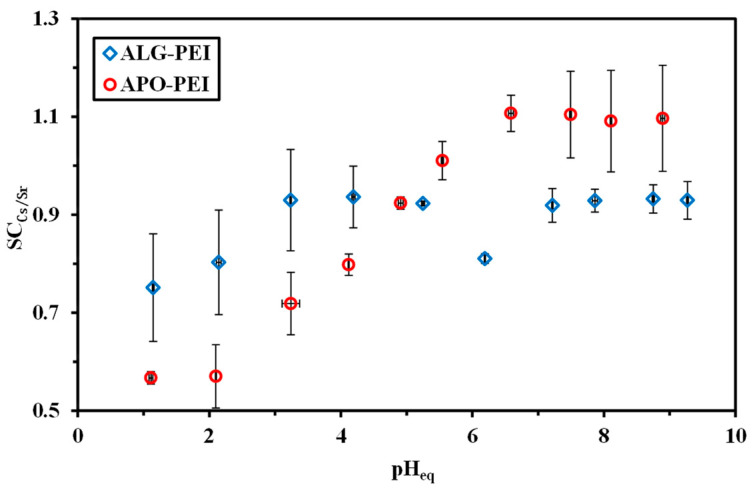
Effect of the pH on the selectivity coefficient (SC_Cs/Sr_) using ALG-PEI and APO-PEI sorbents (binary solutions: 0.754 mmol Cs L^−1^ and 1.227 mmol Sr L^−1^; SD: 0.67 g L^−1^; v: 210 rpm; time: 48 h; T: 21 ± 1 °C).

The very similar profiles of sorption capacity vs. equilibrium pH hamper the prediction of the separation of Cs(I) from Sr(II) onto these sorbents while varying the pH. The same experiments were performed with binary solutions (as illustrated in Figure A11, with C_0_ values of 0.754 mmol Cs L^−1^ and 1.227 mmol Sr L^−1^, meaning non-equimolar conditions with an excess of strontium). The selectivity coefficient (SC_Cs/Sr_) is defined as

(1) $$SC_{Cs/Sr}=\frac{D\left(Cs\right)}{D\left(Sr\right)}=\frac{q_{eq,Cs}\times C_{eq,Sr}}{C_{eq,Cs}\times q_{eq,Sr}}$$

where C_eq_ (mmol L^−1^) and q_eq_ (mmol g^−1^) are the residual concentration and sorption capacity at equilibrium (for the two metal ions), respectively.

The figure shows that under favorable pH conditions for the sorption of Cs(I) and Sr(II) (i.e., higher than pH 4), SC_Cs/Sr_ varies between 0.8 and 1.1. This result confirms the difficulty to separate the two metal ions.

#### 2.2.2. Uptake Kinetics

**Figure 3:**
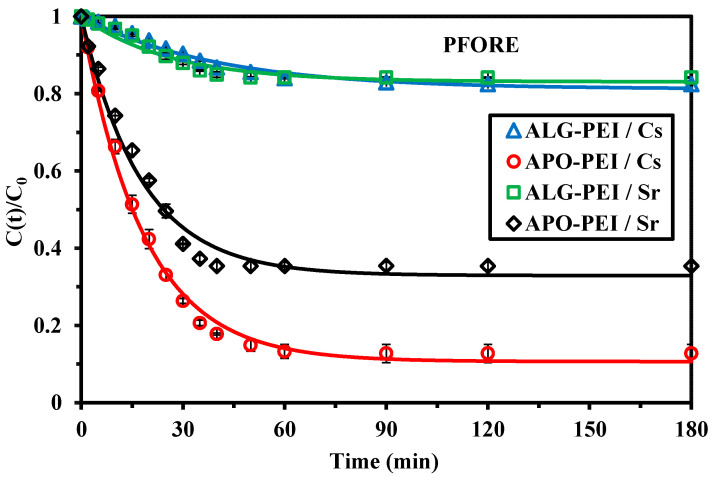
Uptake kinetics for Cs(I) and Sr(II) sorption using ALG-PEI and APO-PEI sorbents (C_0_: 0.760 mmol Cs L^−1^ or 1.188 mmol Sr L^−1^; sorbent dose, SD: 0.67 g L^−1^; v: 210 rpm; pH_0_: 7; T: 21 ± 1 °C).

The kinetics of sorption may be controlled by different mechanisms associated with a resistance to diffusion (bulk, film, and/or intraparticle [57,58]) and to the proper reaction rate (which may be fitted by the pseudo-first (PFORE) and the pseudo-second order rate equations (PSORE) [59]). Table A2 reports the relevant equations for the PFORE and PSORE equation, together with the Crank equation used for simulating the resistance to intraparticle diffusion in a spherical sorbent and under finite volume conditions [60]. Figure 3 shows the kinetic profiles for Cs(I) and Sr(II) sorption using the two sorbents. As expected from Figure 2, the relative residual concentrations are very close for the two metal ions when using ALG-PEI. On the other hand, the largest difference in residual concentrations is observed for APO-PEI (the difference being expanded by the highest concentration of Sr(II), compared with Cs(I)). For the different systems, a contact time of 60–90 min is sufficient for reaching equilibrium. The profiles are characterized by a quasi-linear trend between 0 and ≈30 min of contact, followed by a curved section until equilibrium is reached. The initial slopes are comparable for Cs(I) and Sr(II) with ALG-PEI, while for APO-PEI, the slope is significantly steeper for Cs(I) than for Sr(II).

Table 1 summarizes the parameters of the models applied to the different systems (together with statistical criteria). The PFORE systematically fits the experimental profiles better (highest R^2^ and |AIC| values). It is usually declared that when the ∆(AIC) value between two models is higher than 2, the difference is statistically significant; this is the case here. The PSORE is frequently associated with an uptake driven by chemical sorption; however, as is frequently the case, the conditions raised by Hubbe et al. [61] are not satisfied here for an appropriate conclusion. It is noteworthy that the PFORE also allows the calculated value of the equilibrium sorption capacity (q_eq,1_) to approach closer to the value of the sorption capacity at equilibrium (q_eq,exp_), compared with the relevant PSORE value (q_eq,2_): the overestimation is less than 7.5%. The sorption capacity at equilibrium is systematically higher for APO-PEI than for ALG-PEI (by four-to-five-fold). This presents additional evidence of the beneficial effect of functionalization.

The apparent rate coefficient is also increased after functionalization by a factor of 2.5 and 1.49 for Cs(I) and Sr(II), respectively. The differences are not very marked between the two metals; at least not enough to expect the possibility of using the kinetic criterion for this separation (with the initial concentration being higher for Sr(II), the driving force is stronger, which, in turn, minimizes the significance of rate coefficient differences). The effective diffusivity coefficient (i.e., D_e_, m^2^ min^−1^) can be approached using the Crank equation. The poorer fit (compared with the PFORE) means that the values should be considered as indicative. Apparently, the effective diffusivity is slightly higher for ALG-PEI compared with APO-PEI, despite the weaker textural properties. For Cs(I), the D_e_ value ranges between 4.37 × 10^−9^ and 1.58 × 10^−9^ m^2^ min^−1^, which is two orders of magnitude lower than the self-diffusivity of Cs(I) in water (i.e., 1.23 × 10^−7^ m^2^ min^−1^) [62]. This is of the same order of magnitude as the values reported by Tsai et al. [26] for Cs(I) sorption onto a PVA/alginate/ferric hexacyanoferrate composite (i.e., 9.6 × 10^−9^ m^2^ min^−1^). The effective diffusivity of Sr(II) in the sorbents ranges between 1.58 × 10^−9^ and 3.81 × 10^−9^ m^2^ min^−1^, which is only one order of magnitude lower than the self-diffusivity of Sr(II) in water (i.e., 4.75 × 10^−8^ m^2^ min^−1^) [62]. The reduced effect in diffusivity (compared with self-diffusivity) for Sr(II) vs. Cs(I) can be correlated with the difference in the ionic radius of their hydrated forms: 1.88 Å for Cs(H_2_O)_12_^+^ vs. 1.26 Å for Sr(H_2_O)_8_^2+^ [63].

**Figure A12:**
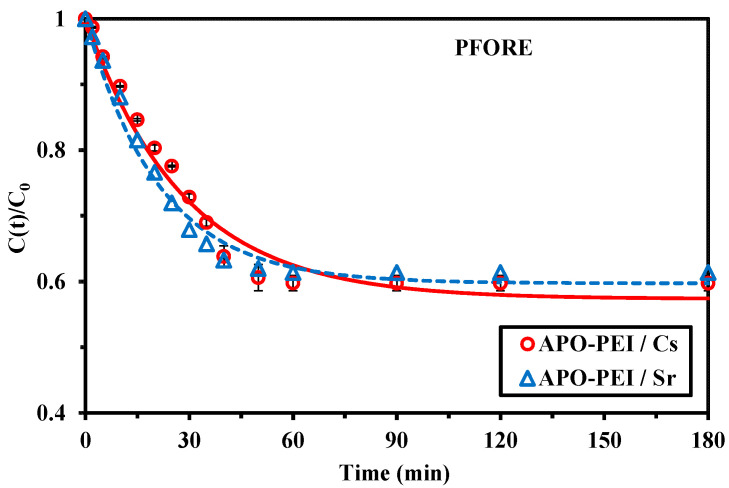
Uptake kinetics for Cs(I) and Sr(II) sorption using APO-PEI sorbents from binary solution (C_0_: 0.751 mmol Cs L^−1^ and 1.148 mmol Sr L^−1^; sorbent dose, SD: 0.67 g L^−1^; v: 210 rpm; pH_0_: 7; T: 21 ± 1 °C).

Figure A12 compares the Cs(I) and Sr(II) concentration decays for APO-PEI sorbent while processing the sorption test from binary solutions. The kinetic curves overlap. The apparent rate coefficient is slightly higher for Sr(II) than for Cs(I) (0.0352 vs. 0.0468 min^−1^), which is consistent with the slightly steeper initial slope in Figure A12. It is noteworthy that these values are slightly lower than the values obtained for mono-component solutions. The maximum sorption capacities are lower than the values obtained in mono-component solutions (≈halved); however, the cumulative equilibrium sorption capacity q_eq,exp,T_ reaches 1.12 mmol g^−1^, which is almost equivalent to the sorption capacity for Sr(II) sorption in mono-component solution (i.e., 1.15 mmol Sr g^−1^). A similar conclusion can be raised concerning the calculated values of the total sorption capacity at equilibrium (i.e., 1.17 mmol g^−1^ vs. 1.19 mmol Sr g^−1^). Cs(I) competes with Sr(II) for occupying the same sorption sites.

#### 2.2.3. Sorption Isotherms

**Figure 4:**
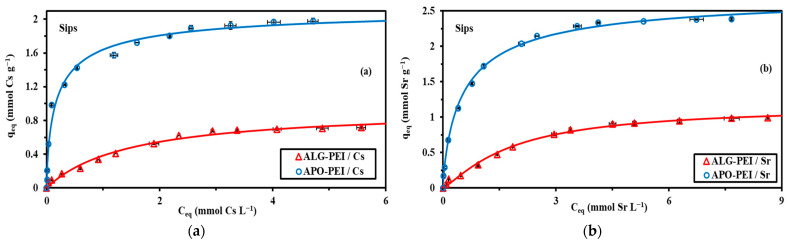
Sorption isotherms for Cs(I) (**a**) and Sr(II) (**b**) using ALG-PEI and APO-PEI sorbents: modeling with Sips equation (pH_0_: 7; C_0_: 0.08–6.11 mmol Cs L^−1^ or 0.12–9.32 mmol Sr L^−1^; SD: 0.67 g L^−1^; v: 210 rpm; time: 48 h; t: 21 ±1 °C).

The sorption isotherms allow the evaluation of the maximum sorption capacity of the sorbents, as well has their affinity for the reactive groups. The plot of sorption capacity (q_eq_, mmol g^−1^) vs. residual solute concentration (C_eq_, mmol L^−1^) represents the distribution of the solute at equilibrium between the two phases (Figure 4). The profiles for the two sorbents and for the two metal ions are similar: an initial curved section (initial slope being correlated to the affinity of the sorbent for the solute) followed by a saturation plateau (representative of the maximum sorption capacity). The comparison of initial slopes and saturation capacities for both Cs(I) (Figure 4a) and Sr(II) (Figure 4b) confirms the strong enhancement of sorption performance. With functionalization, the maximum sorption capacities increase by 2.77 times for Cs(I) and 2.41 times for Sr(II).

**Figure A13:**
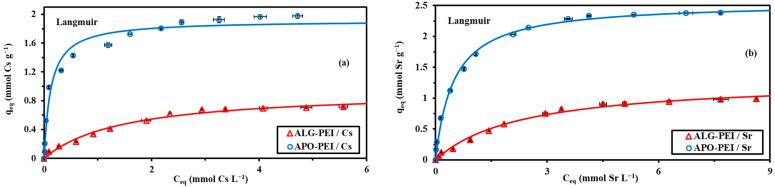
Sorption isotherms for Cs(I) (**a**) and Sr(II) (**b**) using ALG-PEI and APO-PEI sorbents: modeling with the Langmuir equation (pH_0_: 7; C_0_: 0.08–6.11 mmol Cs L^−1^ or 0.12–9.32 mmol Sr L^−1^; SD: 0.67 g L^−1^; v: 210 rpm; time: 48 h; t: 21 ± 1 °C).

Different equations have been designed for modeling the sorption isotherms (Table A3). Table 2 summarizes the parameters of these models. The shape of the isotherms allows the prediction that the Freundlich equation (which is a power-type function) cannot simulate the asymptotic trend of experimental profiles. The Freundlich model supposes the material as being heterogeneous, with sorption operating with possible interactions between sorbed molecules. The Langmuir equation corresponds to a mechanistic equation associated with a homogeneous distribution of sorption energies, without interaction between sorbed molecules and with monolayer accumulation. The maximum sorption capacity corresponds to the sorption capacity at saturation of the monolayer (q_m,L_), while the affinity coefficient (b_L_) is correlated to the initial slope of the isotherm. The Sips equation combines the Langmuir and the Freundlich equations; with the introduction a third-adjustable parameter, the mathematical equation may fit the experimental profile easier, at the expense of a loss in physical significance (compared with the Langmuir equation). Table 2 shows that the Sips equation generally gives the best fit of experimental curves (with the exception of Cs(I) sorption onto ALG-PEI, where the Langmuir equation is more appropriate). In Figure 4, the solid lines represent the Sips fits. Figure A13 shows the fitting of sorption isotherms with the Langmuir equation. The Temkin and Dubinin–Radushkevich (D–R) equations have been also tested. The Temkin equation supposes that the energy of adsorption varies exponentially with the coverage of sorbent surface [64]. The D–R equation, initially designed for gas/solid sorption (especially for mesoporous solids), was extended to liquid/solid sorption. While the Langmuir equation was adapted for describing the sorption into mesoporous and macroporous sorbents with successive adsorption layers, the D–R concept is based on the filling of porous space [65]. Table 2 shows that these two models give poor fits of sorption isotherms.

The phosphorylation of ALG-PEI beads increases the maximum sorption capacities, such as q_m,L_ values for the Langmuir equation and q_m,S_ values for the Sips equation (which is almost doubled, Table 2). The increases in maximum (experimental) sorption capacities reach up to 1.265 mmol Cs g^−1^ and 1.398 mmol Sr g^−1^. These values can be compared with the increase in the number of reactive groups on the sorbent based on elemental analysis (i.e., 0.23 mmol N g^−1^, 0.96 mmol O g^−1^, and 0.73 mmol P g^−1^). The affinity coefficient (i.e., b_L_) also strongly increases from 0.675 to 8.12 L mmol^−1^ for Cs(I) and from 0.430 to 2.05 L mmol^−1^ for Sr(II). The beneficial effect is significantly more marked for Cs(I) (12-fold increase) than for Sr(II) (less than five-fold increase). The major reactive groups present on the sorbent are the amine groups, which are considered soft bases according the hard and soft acid and base theory (HASB [66]), while phosphate and carboxylic groups are classified among the strong bases. On the other hand, Cs(I) is a softer acid compared with Sr(II), which is part of hard acids. According HSAB principles, cesium has more affinity for soft bases, such as amine groups, contrary to strontium readily bound to hard acids, such as carboxylate [67] and phosphonate groups [68,69]. The exact proportion of free reactive groups (especially amine and carboxylate groups) available for metal binding (controlled by steric hindrance and non-engaged in the structuration of ALG-PEI, and chemical modification) is difficult to evaluate.

APO-PEI bears additional phosphonate reactive groups (compared with pristine ALG-PEI); this chemical modification clearly introduces heterogeneities at the surface of the sorbents with functional groups that have a different affinity for the metal ions. This kind of situation can be addressed (in terms of isotherm modeling) using the so-called Langmuir dual site equation (LDS) [70]:

(2) $$q_{eq}=\frac{q_{m,L,1}\times b_{L,1}\times C_{eq}}{1+b_{L,1}\times C_{eq}}+\frac{q_{m,L,2}\times b_{L,2}\times C_{eq}}{1+b_{L,2}\times C_{eq}}$$

where C_eq_ (mmol L^−1^) is the residual concentration; q_eq_ (mmol g^−1^) is the sorption capacity at equilibrium; q_m,L,1_ and q_m,L,2_ are the maximum sorption capacities (mmol g^−1^) for sites 1 and 2, respectively; and b_L,1_ and b_L,2_ are the affinity coefficients (L mmol^−1^) for sites 1 and 2, respectively.

**Figure A14:**
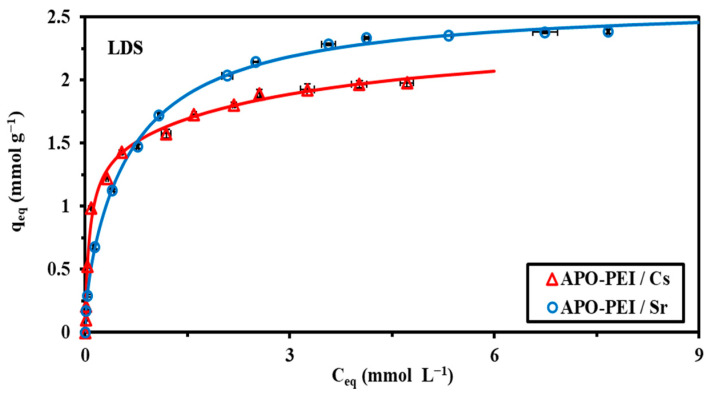
Modeling of Cs(I) and Sr(II) sorption isotherms (onto APO-PEI beads) using the Langmuir dual site model (LDS) (for experimental conditions, see Figure A12).

Figure A14 shows the fitting of Cs(I) and Sr(II) sorption isotherms onto APO-PEI using the LDS equation with the parameters summarized in Table A4. The LDS model gives a good fit of experimental profiles. It is noteworthy that the contributions of sites 1 and 2 significantly differ for Cs(I) and Sr(II). This was anticipated because Cs(I) can be considered a soft acid having a greater affinity for amine groups (compared to O-bearing functional sites). This is the opposite for Sr(II) (hard acid): the contribution of phosphonate groups for strontium binding is significantly enhanced.

**Figure A15:**
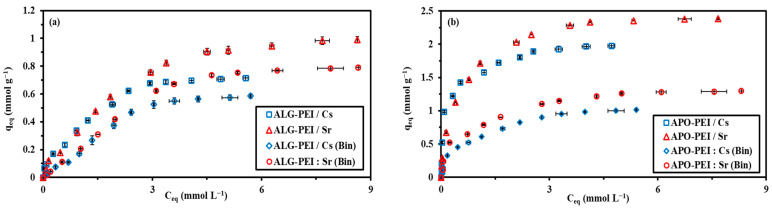
Cs(I) and Sr(II) sorption isotherms from binary solutions using ALG-PEI (**a**) and APO-PEI (**b**) sorbents (compared with isotherms from mono-component solutions—C_0_: 0.07–6.11 mmol Cs L^−1^ and 0.11–9.23 mmol Sr L^−1^, with Sr/Cs molar ratio ≈1.5; pH_0_: 7; SD: 0.67 g L^−1^; v: 210 rpm; T: 21 ± 1 °C).

Binary sorption isotherms were obtained and compared with those obtained in mono-component solutions. Figure A15a shows for ALG-PEI that the presence of Cs(I) reduces the sorption of Sr(II) by 0.20 mmol Sr g^−1^ (i.e., 20%); the presence of Sr(II) reduces Cs(I) sorption capacity by 0.13 mmol Cs g^−1^ (i.e., 18%). The presence of any metal has a similar decreasing effect (around 20%) on the sorption of the complementary metal. The total sorption capacity q_T_(ALG-PEI) reaches 1.30 mmol g^−1^; this is higher than the maximum sorption capacities reached in mono-component solutions (i.e., 0.715 mmol Cs g^−1^ and 0.992 mmol Sr g^−1^). The simultaneous presence of soft and hard acid metals allows the saturation of different reactive groups. Figure A15b shows similar profiles for APO-PEI: the presence of the competitor ion decreases the sorption capacity of the other ion by 45–49%. The cumulative sorption capacity q_T_(APO-PEI) reaches 2.31 mmol g^−1^; this is slightly lower than the maximum sorption capacity obtained for Sr(II) in mono-component solutions (i.e., 2.39 mmol Sr g^−1^). Similar trends were reported for the sorption of Cs(II) and Co(II) using nano-cryptomelane [71].

Table A5 and Table A6 compare the sorption properties of ALG-PEI and APO-PEI with a series of alternative sorbents for Cs(I) and Sr(II) recovery, respectively. Taking into account both the equilibrium (maximum sorption capacity) and the kinetic characteristics, APO-PEI is part of the most efficient sorbents for both Cs(I) and Sr(II); the less favorable parameter is most likely the affinity coefficient, which is less attractive than some of these alternative sorbents. However, APO-PEI appears to be a good compromise in terms of equilibrium and kinetic performances, especially for Sr(II) sorption.

#### 2.2.4. Sorption Selectivity

**Figure 5:**
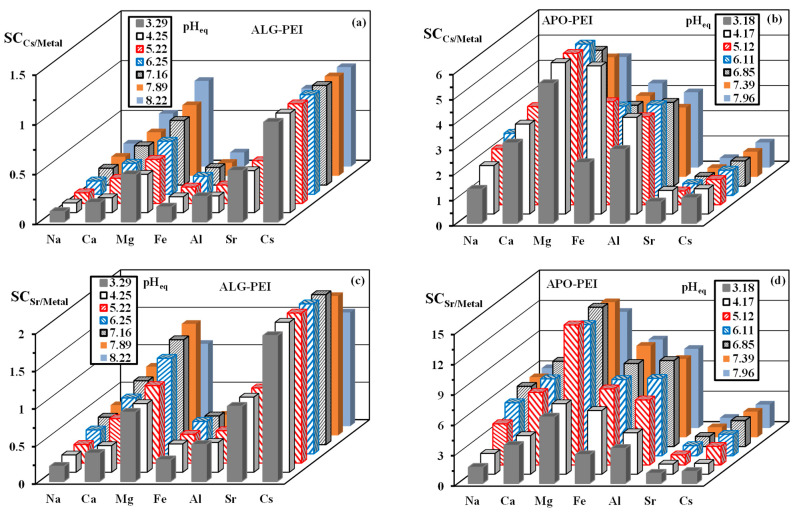
Metal sorption from multicomponent solutions using ALG-PEI (**a**,**c**) and APO-PEI (**b**,**d**) sorbents: effect of pH_eq_ on SC_Cs/metal_ (**a**,**c**) and SC_Sr/metal_ (**b**,**d**) (C_0_, mmol L^−1^: 0.883 Na(I), 0.530 Ca(II), 1.020 Mg(II), 0.881 Fe(III), 0.875 Al(III), 1.088 Cs(I), and 0.959 Sr(II); time: 24 h; v: 210 rpm; T: 21 ± 1 °C; SC_Cs/Cs_ and SC_Sr/Sr_ are mentioned as internal standard = 1).

In order to evaluate the selectivity of the sorbents for Cs(I) and Sr(II), complementary experiments were performed in multi-component solutions (at equimolar concentrations of ≈1 mmol L^−1^, except for Ca(II) at 0.53 mmol L^−1^), at different pH values. Figure 5 reports for both ALG-PEI and APO-PEI the evolution of the selectivity coefficients with pH_eq_ for Cs(I) and Sr(II) against other metal ions present in the multi-component solutions. First, the comparison of the scales of the figures immediately demonstrates that the phosphorylation of ALG-PEI allows the increase of the SC value by four-fold for Cs(I) and up to seven-fold for Sr(II). The grafting of phosphonate moieties not only increases the sorption capacity but also enhances the preference of the sorbent for Cs(I) and Sr(II). The stronger effect for selective Sr(II) recovery compared with Cs(I) can be explained by the increase in the density of hard base reactive groups (phosphonate), which have a higher reactivity for hard acids (such as Sr(II)) vs. softer acid (such as Cs(I)). In most cases, the selectivity increases with the pH, which is most likely associated with the deprotonation of reactive groups (linked with pH_PZC_).

ALG-PEI is poorly selective for Cs(I): the SC_Cs/metal_ values are systematically lower than 1 (Figure 5a). This means that the sorbent will preferentially bind competitor metals. This is confirmed by the sorption capacity at pH_eq_ that does not exceed 0.021 mmol Cs g^−1^ (which is about 10 times lower than the value obtained in mono-component solutions). The loss in sorption capacity is somewhat less marked for Sr(II) (0.038 mmol Sr g^−1^ vs. ≈0.25 mmol Sr g^−1^). Under the most favorable pH conditions, the SC values decrease, according to Figure 5a,c, as follows:

For Cs(I): Mg(II) > Sr(II) > Ca(II) > Al(III) > Fe(III) > Na(I);

For Sr(II): Cs(I) > Mg(II) > Ca(II) > Al(III)> Fe(III) > Na(I).

**Figure A16:**
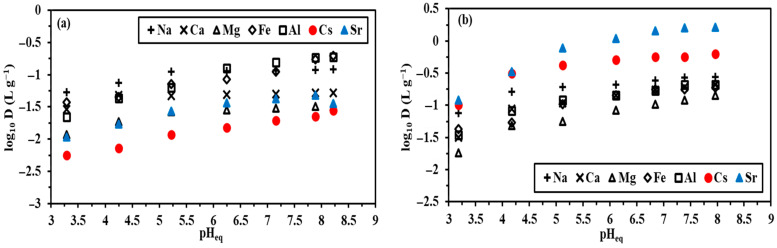
Metal sorption from multicomponent solutions using ALG-PEI (**a**) and APO-PEI (**b**) sorbents: effect of pH_eq_ on log_10_D vs. pH_eq_ (C_0_, mmol L^−1^: 0.883 Na(I), 0.530 Ca(II), 1.020 Mg(II), 0.881 Fe(III), 0.875 Al(III), 1.088 Cs(I), and 0.959 Sr(II); time: 24 h; v: 210 rpm; T: 21 ± 1 °C).

The ranking of sorbent affinity can be also visualized in Figure A16a, in which the decimal logarithm of the distribution ratio (D) is plotted against pH_eq_, as follows:

Al(III) ≈ Fe(III) ≈ Na(I) > Ca(II) ≈ Sr(II) > Mg(II) > Cs(I).

The affinity of a fixed reactive group for a metal cation is controlled by its electronegativity, valence or charge, radius, and/or polarity. This concept was theorized by Pearson [66] in the HSAB principles: hard metal ions react easily and preferentially with hard base ligands (having highly electronegative donor atoms) with the formation of ionic interactions. Reciprocally, soft metal ions favorably react with soft bases (having a lower electronegativity, such as those bearing N-donor atoms) to form coordination bonds [56]. Amine groups can bind soft heavy metal cations at neutral pH and metal anions in acidic solutions (protonated). On the other hand, for sorbents bearing acidic groups, such as carboxylic acid (for example alginate in ALG-PEI) or phosphonic acid groups (for example in APO-PEI), the binding mechanism may involve electrostatic interactions and/or chelation, depending on the pH. Therefore, the pH can modulate the affinity of the sorbents for target metals by changing the major sorption mechanisms. This modulation, in turn, affects the selectivity of sorption. Indeed, under specific conditions, where sorption occurs through the electrostatic attraction and ion-exchange mechanism, uptake will be controlled by parameters such as the ionic charge of the metal ions (between mono-, di-, and trivalent cations). In contrast, when the pH conditions promote binding by the chelation mode, the sorption will be affected by the ionic size of hydrated metal ions (the spatial arrangement and the complexation constant of the analogous ligand for target metals). In fact, all selected metal ions are hard acids (except Fe(III), which belongs to borderline metals [72]. The metal ions can be ranked based on the Pauling electronegativity scale according to:

Cs(I) (0.79) < Na(I) (0.93) < Sr(II) (0.95) < Ca(II) (1.00) < Mg(II) (1.31) < Al(III) (1.61) < Fe(III) (1.830).

Additionally, they can be ranked in terms of ionic size (hydrated species, Å) according to:

Al(III) (0.535) < Fe(III) (0.645) < Mg(II) (0.72) < Na(I) (1.02) < Ca(II) (1.12) < Sr(II) (1.26) < Cs(I) (1.88).

At pH_eq_ ≈ 3.3 and for ALG-PEI, the ranking of D values follows the series:

Na(I) > Fe(III) > Ca(II) > Al(III) > Mg(II) ≈ Sr(II) >> Cs(I).

At pH_eq_ ≈ 8.2 and for ALG-PEI, the ranking of D values follows the series:

Fe(III) ≈ Al(III) > Na(I) >> Ca(II) > Sr(II) > Mg(II) > Cs(I).

At pH_eq_ ≈ 3.2 and for APO-PEI, the ranking of D values follows the series:

Sr(II) > Cs(I) > Na(I) > Fe(III) > Al(III) > Ca(II) > Mg(II).

At pH_eq_ ≈ 8.0 and for APO-PEI, the ranking of D values follows the series:

Sr(II) >> Cs(I) >> Na(I) > Ca(II) > Al(III) > Fe(III) >> Mg(II).

These differences in the capacity of the sorbents to bind metal ions (concentrating effect measured through D values) when pH changes can be assigned to the variations in the binding mechanisms and their metal-dependent effect. This is summarized in Table A7.

The patterns are completely different for APO-PEI (Figure 5b,d and Figure A16b). The SC_Cs/metal_ and SC_Sr/metal_ values are systematically larger than 1, marking the preference of the functionalized sorbent for Cs(I) and Sr(II) as follows, according to decreasing selectivity:

For Cs(I): Mg(II) > Fe(III) > Ca(II) ≈ Al(III) > Na(I) > Sr(II);

For Sr(II): Mg(II) > Fe(III) > Al(III) ≈ Ca(II) > Na(I) > Cs(I).

The decimal logarithmic plot of the distribution ratio vs. pH_eq_ (Figure A15b) confirms the reversed trend (compared with ALG-PEI):

Sr(II) >> Cs(I) >> Na(I) > Al(III) ≈ Ca(II) ≈ Fe(III) > Mg(II).

Despite the preference of APO-PEI for Sr(II) and Cs(I) in multi-component solutions, the sorption capacities in the presence of competitor ions are reduced compared to the levels reached in mono-component solutions. For Sr(II), the sorption capacity decreases from 1.0–1.1 mmol Sr g^−1^ to 0.40 mmol Sr g^−1^, which is less than the loss observed for Cs(I), from 1.0–1.1 mmol Cs g^−1^ to 0.32 mmol Sr g^−1^. These values of sorption capacities can be compared with the levels of sorption reached in binary solutions (Section 2.2.3., i.e., ≈0.59 mmol Sr g^−1^ and ≈0.39 mmol Cs g^−1^). The presence of large amounts of other competitor metal ions weakly decreases the sorption of target metals (compared with the case of binary solutions).

**Figure A17:**
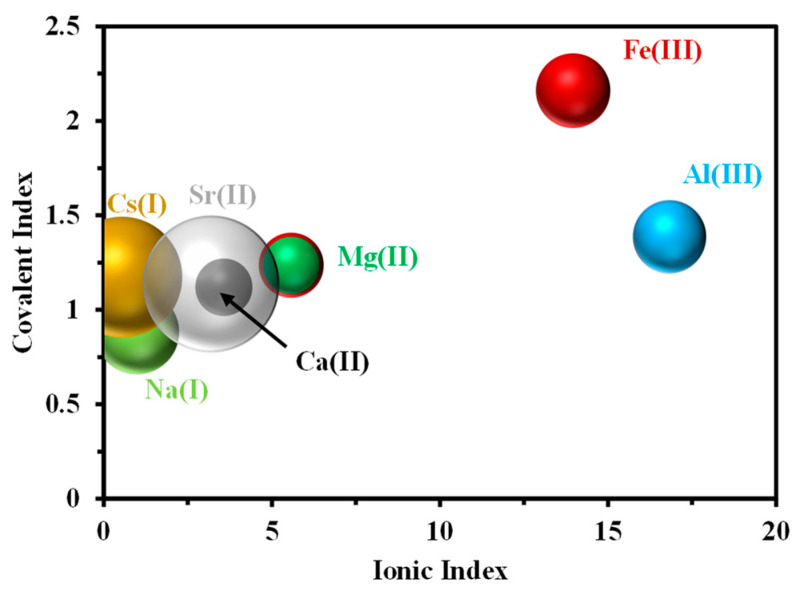
Sorption capacity (proportionality given by the size of the bubble) vs. the positioning of individual metals in the covalent index/ionic index space (data collected from [1,63,100]).

Figure A17 checks if the sorption capacity of individual metals (from multi-component solutions) can be correlated to their covalent index (CI, defined as X_m_^2^ × r, where X_m_ is the Pauling electronegativity and r is the ionic radius of hydrated species) and the ionic index (II, defined as Z^2^/r, where Z is the charge of metal ion). In the classification of Lewis acids, Pearson [66] and Nieboer and Richardson [72] gave a conflicting ranking of Cs(I) (soft and hard, respectively); the positioning of the Cs(I) in Figure A17 means that Cs(I) can be considered a hard acid with a higher affinity for O-bearing ligands. The respective positioning of the different metals investigated in this study (hard/intermediary classes) are difficult to correlate with their affinity for the sorbents. This global criterion is not sufficient for explaining the remarkable increase in selectivity after phosphorylation. It is not possible to correlate the sorption capacity to the metal positioning in the CI/II space. Despite the very close physicochemical characteristics of the couples (monovalent: Cs(I), Na(I); and divalent: Sr(II), Ca(II)), the APO-PEI sorbent shows a marked preference for target metal ions. For a given ionic charge (+1 or +2), the sorption capacity increases with a lower electronegativity and a higher ionic hydrated radius. The configuration of these hydrated metal ions in water are grouped as follows: an octahedron structure for Na(I), Mg(II), Fe(III), and Al(III); a square antiprism for Ca(II) and Sr(II); while Cs(I) supports a 12-coordination structure [63]. It is, thus, difficult to correlate strictly the sorption properties in multi-component solutions (and consequently the selectivity) with specific physicochemical criteria.

#### 2.2.5. Metal Desorption and Sorbent Recycling

**Figure A18:**
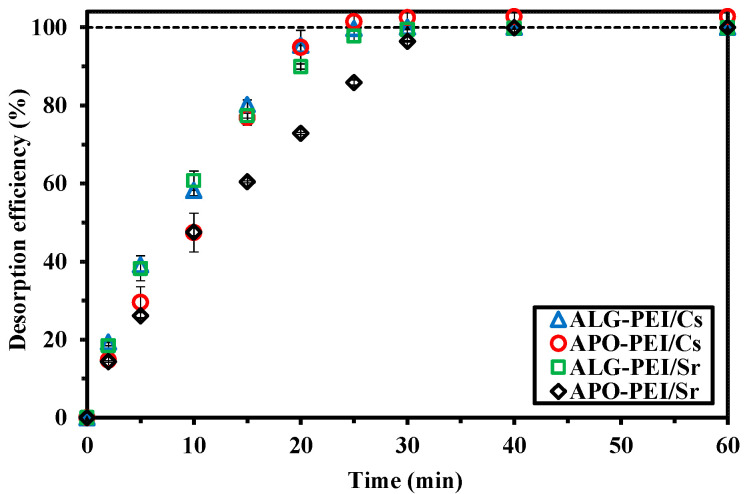
Cs(I) and Sr(II) desorption kinetics for ALG-PEI and APO-PEI sorbents: case of mono-component systems (metal-loaded samples collected at equilibrium from the relevant kinetics; SD: 2.67 g L^−1^; eluent: 0.2 M HNO_3_; v: 210 rpm; T: 21 ± 1 °C).

**Figure A19:**
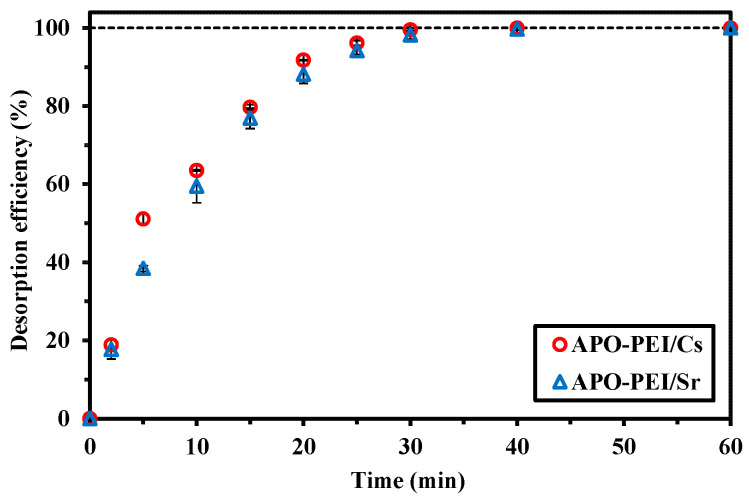
Cs(I) and Sr(II) desorption kinetics for APO-PEI sorbent: case of binary systems (metal-loaded samples collected at equilibrium from the relevant kinetics; SD: 2.67 g L^−1^; eluent: 0.2 M HNO_3_; v: 210 rpm; T: 21 ± 1 °C).

The strong impact of pH on Cs(I) and Sr(II) sorption provides a good direction for selecting the conditions of sorption reversibility and metal desorption. Hence, in acidic solutions, the sorption of target metal ions is negligible: the protonation of reactive groups limits metal binding. Acidic solutions may be efficient for eluting these metal ions. Thus, selecting an acid with which a counter anion can form a stable complex with high solubility is favorable. Using organic ligands (such as EDTA) would be an alternative solution; however, post-treatment of the eluate would be more complex. HNO_3_ (0.2 M) was chosen for eluting Cs(I) and Sr(II). Figure A18 compares the desorption kinetics for the different systems. The profiles of desorption are roughly superposed, and full metal desorption is achieved in 30 min. Figure A19 shows the same desorption process applied to APO-PEI sorbent loaded with binary solution. The same trends are observed for the release of Cs(I) and Sr(II) in terms of kinetics and final desorption efficiency. Nitric acid (0.2 M) is highly efficient for the recovery of target metals.

The possibility to reuse the sorbent after elution is of critical importance. FTIR analyses already demonstrated that after five cycles of reuse, the chemical structure of the sorbents was very close to original materials (see Section 2.1.2.). Table 3 confirms these trends through the comparison of sorption and desorption efficiencies for five successive cycles. The complete desorption efficiencies are maintained along the five cycles for both ALG-PEI and APO-PEI sorbents. The sorption efficiency progressively decreases with recycling. For both Cs(I) and Sr(II), the sorption efficiency of ALG-PEI decreases by about 11.8% at the fifth cycle. On the other hand, the decrease in metal sorption does not exceed 1.7% with the APO-PEI sorbent. This is another advantage brought by the functionalization of pristine sorbent. The APO-PEI sorbent demonstrates remarkable stability in sorption and desorption performance, at least under the selected experimental conditions.

### 2.3. Cs(I) and Sr(II) Sorption from Seawater

**Figure 6:**
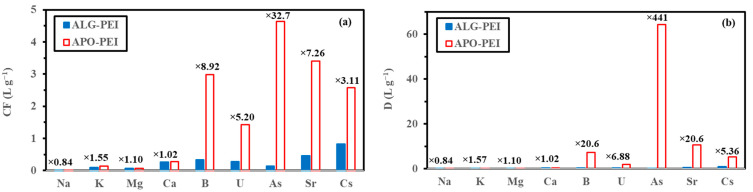
Concentration factor (C = q_eq_/C_0_, L g^−1^) (**a**) and distribution ratio (D = q_eq_/C_eq_, L g^−1^) (**b**) for selected elements for the treatment of the seawater sample using ALG-PEI and APO-PEI sorbents (for initial concentrations, see Table A8; SD: 0.2 g L^−1^; pH_0_: 7.59; pH_eq_: 7.51; time: 24 h; v: 210 rpm; T: 21 ± 1 °C; individual numbers represent the enhancement factor associated with the functionalization of ALG-PEI sorbent).

**Figure A20:**
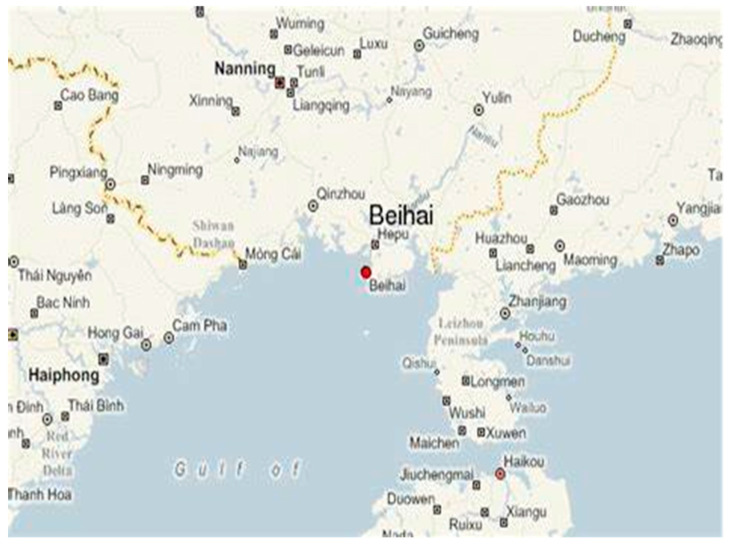
Location of sample collection (Beihai, China).

The sorption of Cs(I) and Sr(II) may be strongly hindered by a harsh environment. The design of this functionalized sorbent was driven by potential application in the removal of radionuclides from seawater (following the Fukushima Daiichi accident). Although this specific experiment was not addressed in this manuscript, the removal of natural elements from real seawater samples is considered hereafter. A seawater sample was collected from Beihai Bay (China, Figure A20). Table A8 collects the concentrations of a series of metal ions. Four major elements are present, with concentrations ranging between 11 and 500 mmol L^−1^ (Na(I) > Mg(II) > K(I) > Ca(II)). On the other hand, Sr(II) and Cs(I) are parts of the trace elements (with concentrations as low as 49 and 2.5 µmol L^−1^, respectively), associated with other elements such as uranium or arsenic. This means that major elements are in huge excess compared with the target metals (several orders of magnitude: from 4600-fold for Ca(II) to 200,000-fold for Na(I)). The sorption tests show that the sorbents exhibit a significant enrichment effect on trace elements, despite the huge excess of major elements. Based on the very low concentration of trace elements, providing the sorption capacities would not be meaningful. Figure 6 shows the concentration factors (CF, defined as q_eq_/C_0_) and distribution ratios for selected elements and for the two sorbents. The figure also shows the enhancement factor resulting from the phosphorylation of ALG-PEI: APO-PEI beads, demonstrating that the concentration factor increases three times for Cs(I) and seven times for Sr(II).

**Figure A21:**
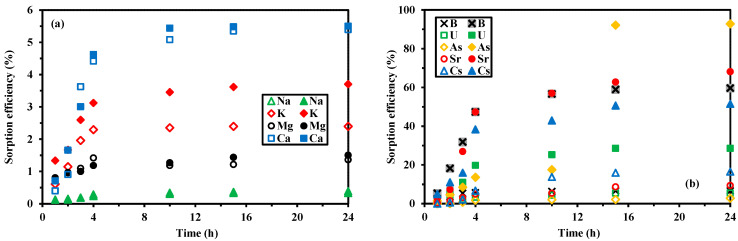
Time evolution of sorption efficiency for major elements (**a**) and trace elements (**b**) using ALG-PEI (empty symbols) and APO-PEI (filled symbols) sorbents (initial concentrations see Table A8; SD: 0.2 g L^−1^; pH_0_: 7.59; pH_eq_: 7.51; v: 210 rpm; T: 21 ± 1 °C).

Figure A21 compares the kinetics of sorption (given as sorption efficiency) for major elements (Figure A21a) and trace elements (Figure A21b) using both ALG-PEI and APO-PEI. The sorption of major elements remains below 6%, regardless of the sorbent. More interesting is the comparison of the profiles for trace elements: the phosphorylation of the pristine sorbent significantly increases the sorption of trace elements up to 51% for Cs(I) and 68% for Sr(II), compared with ALG-PEI (i.e., 17% and 10%, respectively), after 24 h of contact.

**Figure A22:**
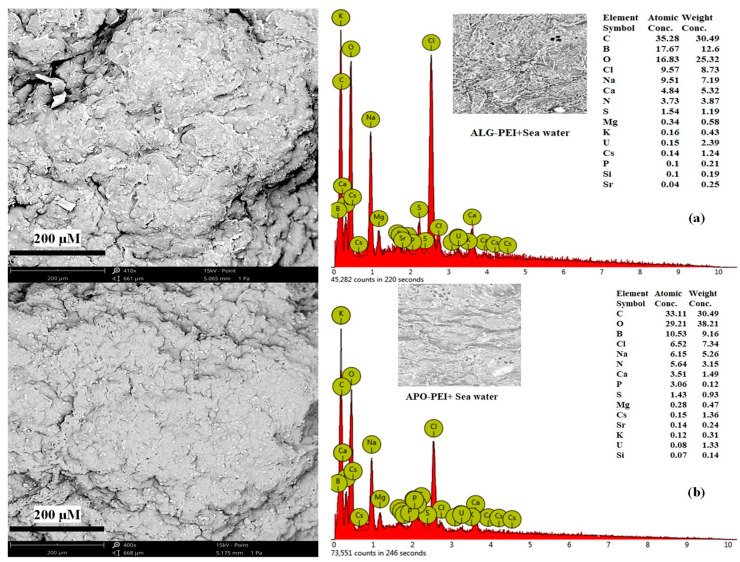
SEM observation (**left** panel) and semi-quantitative EDX analysis (**right** panels) of ALG-PEI (**a**) and APO-PEI (**b**) after the treatment of seawater.

The sorbents exposed to seawater were semi-quantitatively analyzed by EDX. Figure A22 shows the morphological aspects of the sorbents after the treatment of seawater, as well as their surface composition. The surface topography appears smoother than the original materials, with the deposition of small micro-particles (most likely associated with local precipitation of metal ions). The semi-quantitative analysis shows the accumulation of Na and Cl (due to the high concentration of these ions); Sr(II) and Cs(I) are identified (at a low concentration, due to their trace amount in seawater). Despite the high concentration of potassium in seawater, the amount of this element at the surface of the sorbents remains negligible. Among the trace elements considered in this study, uranium appears relatively high in the semi-quantitative analysis (up to 2.39% in weight; 0.15 At.%). It is important to note that the EDX analysis is limited to ≈2 µm in depth. This can explain the apparent inconsistency with the levels of enrichment reported in Figure 6 (which calculate the concentration factors at the macroscale level).

## 3. Conclusions

The main conclusions of this work can be summarized as follows:

- ALG-PEI is efficient for the sorption of both Cs(I) and Sr(II), at a pH close to neutral; however, the efficient phosphorylation of the pristine beads allows, in the same pH domain, a significant improvement of the sorption properties;
- While alginate and amine groups are involved in metal binding in ALG-PEI, phosphonate groups bring additional possibilities for metal binding (especially for Sr(II));
- Under selected experimental conditions, the uptake kinetics, controlled by the pseudo-first order rate equation, is relatively fast: the equilibrium is reached in 60–90 min. Although the resistance to intraparticle diffusion cannot be entirely neglected, the textural properties of the sorbents allowed the contribution of diffusional constraints to be reduced;
- The sorption isotherms are well fitted by the Sips equation (slightly better than the Langmuir equation), with a preference for Sr(II) sorption over Cs(I), especially in the case of APO-PEI beads;
- The functionalization improves the selectivity of the sorbent for target metals against alkali, alkaline-earth, and heavy metals, both in synthetic solutions and complex solutions (as seawater);
- The functionalization also improves the stability in the sorption performances (after five cycles).

These results show the promising perspectives of these materials for the treatment of elements frequently found in the effluents resulting from nuclear accidents. Clearly, some pending questions should be considered before giving a definitive statement on the efficiency against radionuclides in seawater environment. An important question concerns the stability of the sorbent under irradiation. Complementary investigations would be also necessary for dimensioning the system for practical applications, including testing the sorbents in fixed bed reactors [36]; the spherical form and the size of the beads (associated with their textural properties) are favorable but the hydrodynamic conditions (flow rate and residence time) are critical parameters to be considered. Some new perspectives are also opened using alternative conditionings of the polymers such as sponges [73,74].

## 4. Materials and Methods

### 4.1. Materials

The brown algae used in this study, *Laminaria digitata*, was purchased from Setalg (Pleubian, France). Iminodi(methylphosphonic acid) (97%), polyethyleneimine (branched PEI, 50%, *w*/*w* in water), strontium nitrate (Sr(NO_3_)_2_, 99.9%), cesium nitrate (CsNO_3_, 99%), sodium chloride (NaCl, >99%), calcium chloride (≥99.9%), magnesium chloride (MgCl_2_, >98%), sodium hydroxide (NaOH, ≥97.0%), glutaraldehyde (GA, 25% in water), and epichlorohydrin (98%) were supplied by Sigma-Aldrich (Taufkirchen, Germany). Poly ethylene glycol diglycidyl ether (PEGDGE, for enhancing the stability by bead crosslinking of the beads), ferric chloride (FeCl_3_, ≥99.5%), and aluminum chloride (AlCl_3_, 95%) were purchased from Guangdong Guanghua Sci-Tech, Co., Ltd. (Guangdong, China). The other reagents were obtained from Prolabo and used as received.

### 4.2. Synthesis of Sorbents

#### 4.2.1. Synthesis of ALG-PEI

**Scheme 2:**
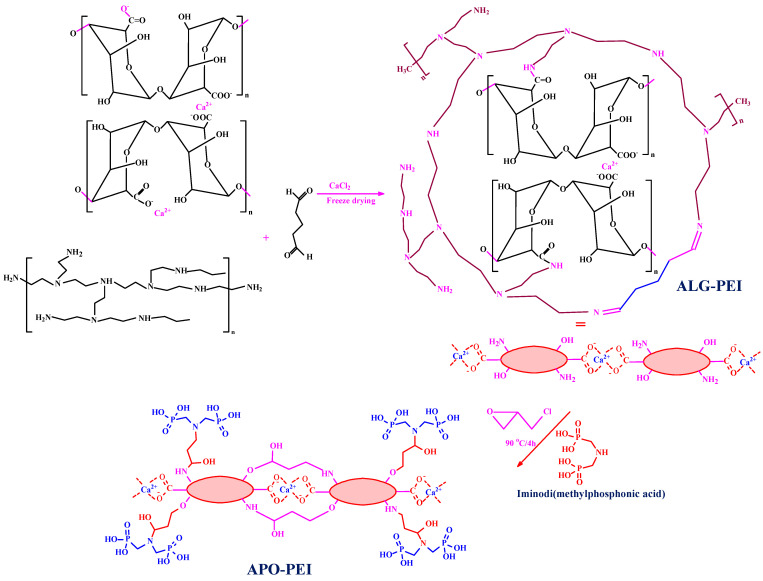
Synthesis procedures for the preparation of ALG-PEI and APO-PEI sorbents.

Twenty grams of algal biomass (*L. digitata*) were dispersed in 600 mL of sodium carbonate solution (1% Na_2_CO_3_, *w*/*w*), the mixture was maintained under agitation for 24 h at 50 °C; this step allowed partial alginate extraction [41]. In a second step, 5 mL of PEI solution were added under agitation to the algal suspension. After homogenization, the mixture was drip-added to 1 L of calcium chloride solution (1% *w*/*w* CaCl_2_ completed with 5 mL of GA solution). Calcium ions react for 24 h through ionotropic gelation with carboxylic groups of alginate (guluronic and mannuronic acid groups). On the other hand, the end aldehyde groups of GA react with the amine groups of PEI for complementary crosslinking (Scheme 2). The interpenetrating networks (resulting from the double crosslinking) contribute to the stability of the hydrogel beads. Finally, the formed beads (ALG-PEI) were freeze-dried (−52 °C, 0.1 mbar) for 24 h.

#### 4.2.2. Synthesis of APO-PEI (Functionalization of ALG-PEI)

For reinforcing the chemical stability of ALG-PEI (which will be submitted to drastic conditions for further modification and during sorption/desorption cycles), a new crosslinking procedure was applied. PEGDGE (3 mL) was added to 5 g of beads (dispersed in 100 mL of isopropanol). The mixture was gently agitated (60 rpm) under reflux (75 °C) for 4 h. The beads were filtered and washed with acetone before being air-dried for 10 h at 50 °C; the yield (after this crosslinking step) reached 6.1 g. Based on the weight variation (assuming that the synthesis does not cause material loss), the reinforcement of the stability of the beads by PEGDGE leads to a reaction yield close to 83%.

For the final phosphorylation of the sorbent, crosslinked beads (5 g) were added to 100 mL of ethanol solution (which contained 8.2 mL of epichlorohydrin and 7.7 g of iminodi(methylphosphonic) acid, 37.4 mmol). The reaction took place in a reactor equipped with a condenser, thermometer, and reflux (at 90 °C) for 4 h. The final functionalized beads were filtered and rinsed with water and acetone then air-dried for 10 h at 50 °C. The yield reached 10.7 g. The functionalization of the beads by grafting of iminodi(methylphosphonic) acid leads to weight variation corresponding to a reaction yield of 55%.

### 4.3. Characterization of Materials

Morphological studies of the surface were performed by scanning electron microscopy (SEM) using a Phenom ProX-SEM (Thermo Fisher Scientific, Eindhoven, The Netherlands). The semi-quantitative elemental characterization was achieved by X-ray analysis (integrated in the Phenom ProX-SEM equipment). The specific surface area (S_BET_) and the porosity were measured using a Micromeritics TriStar II analyzer (Micromeritics, Norcross, GA, USA). Sorbent samples were first swept under N_2_ atmosphere for four hours at 130 °C prior to analysis. The BJH method was used for analyzing the porosity of APO-PEI, while the S_BET_ was calculated from the BET equation. The thermodegradation of the materials was investigated by TG-DTA using an STA-449 F3-Jupiter thermoanalyzer (Netzsch, Gerätebau-HGmbh, Selb, Germany). The analysis was performed under N_2_ atmosphere, with a 10 °C min^−1^ temperature ramp. FT-IR spectra were acquired using an IR-Tracer 100 FTIR spectrometer (Shimadzu, Tokyo, Japan). The samples were first dried at 60–65 °C before being conditioned as KBr pellets. The pH_PZC_ (pH of zero charge) was determined using the pH-drift method [75]. Elemental analysis of dry samples was performed using a Vario-EL cube element analyzer (Elementar Analysensysteme GmbH-Langenselbold, Germany). A series of varying pH solutions (pH_0_, from 1 to 11, using sodium chloride as the background salt) was mixed with the sorbent (at SD: 2 g L^−1^) for 48 h. The final pH (i.e., pH_eq_) was recorded and compared with the initial pH; pH_PZC_ corresponds to pH_0_ = pH_eq_. The pH of the solutions was measured using an S220-seven Compact pH-ionometer (Mettler-Toledo Instruments, Shanghai, China). Cesium concentration was measured using AAS (atomic absorption spectrometry) through Unicam-969 (Thermo Electron Corporation, Waltham, MA, USA). Other elements (i.e., Sr, Fe, As, U, Al, etc.) were determined by an inductively coupled plasma atomic emission spectrometry (ICP-AES) using an ICP-7510 instrument (Shimadzu, Tokyo, Japan). Sodium concentration in solution was measured by flame atomic absorption (FAAS) using an AA-7000 spectrophotometer (Shimadzu, Tokyo, Japan).

### 4.4. Sorption Studies

The sorption tests were performed in batch. A known volume of solution (V, L) at target pH_0_ and given metal concentration (C_0_, mmol L^−1^) was mixed with a known amount of sorbent (m, g; corresponding to SD: m/V, g L^−1^), under agitation (v: 210 rpm). The temperature was set to 21 ± 1 °C. After fixed contact times (for kinetics) or after 48 h (for isotherms and equilibrium experiments), a sample was collected and filtered (using a filter membrane with a pore size of 1.2 µm). The residual metal concentration (C_eq_, mmol L^−1^) was analyzed, while the equilibrium pH was systematically monitored (not adjusted during sorption experiment). The mass balance equation served to calculate the sorption capacity (q_t_ or q_eq_, mmol g^−1^): q = V × (C_0_ − C)/m. Experiments were duplicated and the data presented show average values (with standard deviation bars).

For the study of metal desorption, the tests were also performed in a batch system. The metal-loaded sorbent collected at the end of the study of uptake kinetics was used for investigating the desorption of Cs(I) and Sr(II) by mixing the loaded sorbent (SD: 2.67 or 2 g L^−1^) with the 0.2 M HNO_3_ acid solution for a variable contact time (desorption kinetics) or after 2 h (for sorption/desorption cycles). The amount of metal desorbed was compared to the initial metal loading for calculating the desorption efficiency. Rinsing steps (using demineralized water) were systematically processed between each sorption and desorption step.

Similar procedures were used for investigating sorption properties in binary solutions, and for studying the sorption selectivity. In complex solutions (herein, the seawater sample with a large excess of sodium, potassium, etc.), the batch method was also applied. The experimental conditions are systematically reported in the caption of specific figures.

The modeling of sorption performances was operated using conventional equations for uptake kinetics (see Table A2 in Appendix B) and sorption isotherms (see Table A3). The quality of model fitting was measured using the determination coefficient (i.e., R^2^) and the Akaike information criterion (i.e., AIC, see Table A3). The non-linear regression analysis was used for the determination of model parameters, using Mathematica^®^ software and a proprietary math netbook.

## Figures and Tables

**Table 1:** Uptake kinetics for the sorption of Cs(I) and Sr(II) using ALG-PEI and APO-PEI sorbents: Parameters of the models.

Sorbent				Solution					
				Mono-Element				Binary	
				ALG-PEI		APO-PEI		APO-PEI	
Model	Parameter	Unit	Metal Ion	Cs(I)	Sr(II)	Cs(I)	Sr(II)	Cs(I)	Sr(II)
Experimental	q_eq,exp_	mmol g^−1^		0.200	0.281	0.998	1.15	0.454	0.665
PFORE	q_eq,1_	mmol g^−1^		0.215	0.299	1.02	1.19	0.480	0.693
	k_1_ × 10^2^	min^−1^		2.50	3.77	5.43	5.62	3.52	4.68
	R^2^	-		0.981	0.962	0.996	0.985	0.980	0.987
	AIC	-		−134	−126	−112	−102	−109	−118
PSORE	q_eq,2_	mmol g^−1^		0.279	0.366	1.19	1.38	0.588	0.820
	k_2_ × 10^2^	g mmol^−1^ min^−1^		8.02	10.6	5.55	4.98	6.19	6.50
	R^2^	-		0.962	0.927	0.973	0.953	0.954	0.958
	AIC	-		−125	−118	−84	−85	−98	−101
RIDE	D_e_ × 10^9^	m^2^ min^−1^		4.37	7.00	1.58	3.81	3.38	4.59
	R^2^	-		0.960	0.935	0.969	0.955	0.955	0.964
	AIC	-		−120	−117	−77	−83	−95	−100

q_eq,i_: equilibrium sorption capacity; k_i_: apparent rate coefficient.

**Table 2:** Cs(I) and Sr(II) sorption isotherms using ALG-PEI and APO-PEI sorbents: parameters of the models.

Sorbent				ALG-PEI		APO-PEI	
Model	Parameter	Unit	Metal Ion	Cs(I)	Sr(II)	Cs(I)	Sr(II)
Experimental	q_m,exp_	mmol g^−1^		0.715	0.992	1.98	2.39
Langmuir	q_m,L_	mmol g^−1^		0.950	1.31	1.92	2.55
	b_L_	L mmol^−1^		0.675	0.430	8.12	2.05
	R^2^	-		0.988	0993	0.978	0.997
	AIC	-		−93	−92	−58	−74
Freundlich	k_F_	mmol^1−1/n_F_^ L^−1/n_F_^ g^−1^		0.370	0.406	1.44	1.48
	n_F_	-		2.23	2.15	3.87	3.46
	R^2^	-		0.967	0.963	0.949	0.959
	AIC	-		−80	−69	−45	−43
Sips	q_m,S_	mmol g^−1^		0.947	1.16	2.15	2.75
	b_S_	(mmol L^−1^)^n_S_^		0.678	0.480	3.18	1.54
	n_F_	-		0.996	0.804	1.41	1.24
	R^2^	-		0.988	0.995	0.987	0.998
	AIC	-		−89	−93	−61	−84
Temkin	A_T_	L mmol^−1^		20.2	8.48	159.8	63.72
	b_T_	kJ kg mol^−2^		16.41	10.61	7.90	6.08
	E_T_	kJ mol^−1^		22.94	10.69	3.99	2.55
	R^2^	-		0.924	0.953	0.993	0.983
	AIC	-		−69	−66	−74	−56
D–R	q_m,D–R_	mmol g^−1^		0.728	0.983	1.42	2.28
	k_D–R_ × 10^7^	mol^2^ kJ^−2^		2.16	3.93	0.206	0.693
	E_D–R_	kJ mol^−1^		2.15	1.60	6.97	3.80
	R^2^	-		0.975	0.984	0.972	0.974
	AIC	-		−77	−75	−54	−46

q_m,exp_: maximum sorption capacity (experimental value); q_m,L_ and q_m,S_: maximum sorption capacities for Langmuir and Sips equations, respectively (calculated values); b_L_ and b_S_: affinity coefficients for Langmuir and Sips equations, respectively; n_F_ and n_S_: exponential parameters of the Freundlich and Sips equation (related to sorption intensity), respectively. k_F_: constant related to sorption capacity according to the Freundlich equation. A_T_: Temkin equilibrium constant; b_T_: Temkin equation related to heat of adsorption; E_T_: Temkin energy of sorption: q_m-D–R_: maximum sorption capacity; k_D–R_: D–R constant related to adsorption energy; E_D–R_: characteristic free energy of adsorption.

**Table 3:** Sorbent reuse: sorption (SE, %) and desorption (DE, %) efficiencies for five successive cycles.

Sorbent		ALG-PEI		APO-PEI	
		Operation			
Metal	Cycle	SE	DE	SE	DE
Cs(I)	1	15.8 ± 1.0	100.2 ± 0.5	86.6 ± 0.2	100.4 ± 0.8
	2	15.3 ± 1.0	100.2 ± 0.5	86.4 ± 0.1	100.0 ± 0.4
	3	14.5 ± 1.1	100.2 ± 0.9	86.0 ± 0.4	100.1 ± 0.3
	4	13.9 ± 1.1	99.2 ± 0.0	85.3 ± 0.1	99.9 ± 0.5
	5	13.5 ± 1.4	100.4 ± 0.5	84.9 ± 0.1	100.3 ± 0.4
	Loss fifth/first	11.8	-	1.72	
Sr(II)	Cycle	SE	DE	SE	DE
	1	14.5 ± 0.7	99.6 ± 0.2	60.6 ± 0.5	100.4 ± 0.3
	2	14.0 ± 0.5	100.4 ± 0.7	60.1 ± 0.6	100.1 ± 0.1
	3	13.4 ± 0.8	100.3 ± 0.3	59.8 ± 0.6	100.2 ± 0.2
	4	13.0 ± 1.1	99.8 ± 0.5	59.5 ± 0.6	100.0 ± 0.1
	5	12.4 ± 0.8	100.2 ± 0.3	59.2 ± 0.6	100.1 ± 1.0
	Loss fifth/first	11.7		1.53	

Sorption step—C_0_: 0.755 mmol Cs L^−1^ or 1.18 mmol Sr L^−1^; pH_0_: 7; SD: 0.67 g L^−1^; v: 210 rpm; time: 24 h; T: 21 ± 1 °C. Desorption step—0.2 M HNO_3_ eluent; SD: 2 g L^−1^; v: 210 rpm; time: 2 h; T: 21 ± 1 °C.

## Data Availability

Data may be obtained from the authors.

## Appendix A. Characterization of Sorbents

**Table A1:** Elemental analysis of ALG-PEI and APO-PEI sorbents.

Sorbent	Unit	C	N	H	O	S	P
ALG-PEI	Mass %	38.40	2.28	9.51	36.04	0.53	0.09
	mmol g^−1^	31.97	1.63	94.35	22.53	0.17	0.03
APO-PEI	Mass %	40.05	2.61	10.27	37.55	0.39	2.35
	mmol g^−1^	33.35	1.86	101.89	23.47	0.12	0.76

## Appendix B. Characterization of Sorption Properties (Synthetic Solutions)

### Appendix B.2. Uptake Kinetics

**Table A2:** Equations used for modeling uptake kinetics [58,59].

Model	Equation	Parameters	Ref.
PFORE	$q\left(t\right)=q_{eq,1}\left(1-e^{-k_{1}\;t}\right)$	q_eq,1_ (mmol g^−1^): sorption capacity at equilibrium k_1_ (min^−1^): apparent rate constant of PFORE	[59]
PSORE	$q\left(t\right)=\frac{q_{eq,2}^{2}k_{2}t}{1+k_{2}q_{eq,2}t}$	q_eq,2_ (mmol g^−1^): sorption capacity at equilibrium k_2_ (g mmol^−1^ min^−1^): apparent rate constant of PSORE	[59]
RIDE	$\frac{q\left(t\right)}{q_{eq}}=1-\overset{\infty }{\underset{n=1}{\sum }}\frac{6\alpha \left(\alpha +1\right)\exp\left(\frac{-D_{e}q_{n}^{2}}{r^{2}}t\right)}{9+9\alpha +q_{n}^{2}\alpha^{2}}$ with q_n_ being the non-zero roots of $\tan q_{n}=\frac{3\;q_{n}}{3+\alpha q_{n}^{2}}$ and $\frac{m\;q}{V\;C_{0}}=\frac{1}{1+\alpha }$	D_e_ (m^2^ min^−1^): effective diffusivity coefficient	[60]

m (g): mass of sorbent; V (L): volume of solution; C_0_ (mmol L^−1^): initial concentration of the solution.

### Appendix B.3. Sorption Isotherms

**Table A3:** Equations used for modeling sorption isotherms.

Model	Equation	Parameters	Ref.
Langmuir	$q_{eq}=\frac{q_{m,L}C_{eq}}{1+b_{L}C_{eq}}$	q_m,L_ (mmol g^−1^): sorption capacity at saturation of monolayer b_L_ (L mmol^−1^): affinity coefficient	[77]
Freundlich	$q_{eq}=k_{F}C_{eq}^{1/n_{F}}$	k_F_ (mmol g^−1^)/(mmol L^−1^)^nF^ and n_F_: empirical parameters of Freundlich equation	[78]
Sips	$q_{eq}=\frac{q_{m,S}b_{S}C_{eq}^{1/n_{S}}}{1+b_{S}C_{eq}^{1/n_{S}}}$	q_m,L_ (mmol g^−1^), b_S_ (mmol L^−1^)^nS^, and n_S_: empirical parameters of Sips equation (based on Langmuir and Freundlich equations)	[57]
Temkin	$q_{eq}=\frac{RT}{b_{T}}\ln\left(A_{T}\;C_{eq}\right)$	A_T_ (L mmol^−1^): equilibrium binding capacity; b_T_: Temkin constant related to sorption heat (J kg^−1^ mol^−2^)	[64,79]
D–R	qeq=qDR exp[−(RT ln(1+C0Ceq)EDR)]2	q_DR_ (mmol g^−1^), E_DR_ (kJ mol^−1^)	[65]

Akaike information criterion, AIC [80]:

(A1) $$AIC=N\ln\left(\frac{\sum_{i=0}^{N}{\left(y_{i,exp.}-y_{i,model}\right)}^{2}}{N}\right)+2N_{p}+\frac{2N_{p}\left(N_{p}+1\right)}{N-N_{p}-1}$$

where N is the number of experimental points, N_p_ is the number of model parameters, y_i,exp_., and y_i,model_ is the experimental and calculated value of the tested variable.

**Table A4:** Parameters for the modeling of Cs(I) and Sr(II) sorption isotherms (onto APO-PEI sorbent) using the LDS equation.

Parameter	Unit	Metal Ion	
		Cs(I)	Sr(II)
q_m,L,1_	mmol g^−1^	1.006	2.305
b_L,1_	L mmol^−1^	0.3240	1.427
q_m,L,2_	mmol g^−1^	1.420	0.3206
b_L,2_	L mmol^−1^	15.98	39.28
R^2^	-	0.994	0.999
AIC	-	−67	−88

**Table A5:** Comparison of Cs(I) sorption properties with alternative sorbents.

Sorbent	pH	Time	q_m,exp_.	q_m,L_	b_L_	Ref.
Amberlite IR120	0.5 M HNO_3_	60	0.035	0.041	9.08	[81]
Amino-functionalized MWCNTs	7	30	0.828	0.882	137	[82]
Ionic liquid-impregnated chitosan	2.5	120	0.0188	0.0185	47.0	[83]
Nanoscale Fe/Cu particles	6	60	0.168	0.283	0.241	[84]
Crosslinked tea leaves	7	1440	2.50	2.48	0.280	[85]
Sulfonated hyper-cross-linked polymers	7	60	1.204	1.11	0.93	[86]
Sodium iron titanate (Ti/Fe = 5)	6	120	0.903	0.950	2.53	[87]
La/vinylphosphonate/phenantroline	3.5	60	0.032	0.034	65.1	[43]
Nitrified wood charcoal	7	240	1.02	1.00	141	[88]
Green rust/composite aluminosilicate	12	120	n.d.	0.971	305	[89]
Magnetic chitosan/bone powder composite	5.2	60	0.162	0.179	3.47	[90]
Activated wood charcoal	7	60	0.252	0.268	17.5	[91]
Mesoporous MnO_2_/SBA-15	7	15	0.715	0.789	4.92	[92]
Prussian-blue/hydroxyapatite/alginate *	SW	5	0.178	0.186	8.90	[28]
Functionalized zirconium phosphonate polymer	0.2 M HNO_3_	240	1.14	1.34	3.02	[93]
Magnetite/chitosan/hexacyanoferrate	5	240	0.263	0.259	267	[94]
Hexacyanoferrate/PVA/GO	7	420	0.346	0.359	214	[95]
Cu/Fe(II) hexacyanoferrate/PVA hydrogel	n.d.	n.d.	2.56	2.97	4.12	[23]
ALG-PEI	7	90	0.715	0.950	0.675	This work
APO-PEI	7	90	1.98	1.92	8.12	This work

n.d.: not documented; *: seawater sample spiked with both Cs(I) and Sr(II); time: min; q_m_: mmol Cs g^−1^; b_L_: L mmol^−1^.

**Table A6:** Comparison of Sr(II) sorption properties with alternative sorbents.

Sorbent	pH	Time	q_m,exp_.	q_m,L_	b_L_	Ref.
Ionic liquid-impregnated chitosan	2.5	120	0.017	0.0165	30.8	[83]
Zirconium phosphonate	n.d.	240	1.35	1.35	3.85	[44]
Aminophosphonic-functionalized chitosan	6	60	0.037	0.039	78.0	[96]
Graphene oxide	6	1440	1.20	1.57	0.876	[97]
Sulfonated hyper-cross-linked polymers	7	5	0.856	0.816	57.0	[86]
Crab carapace	n.d.	240	0.038	0.045	11.4	[98]
SrTreat resin	n.d.	30	0.104	0.109	265	[98]
Kurion-TS-G	n.d.	30	0.128	0.230	492	[98]
Dowex HCR-S/S	7	60	1.54	1.91	0.083	[99]
Sodium iron titanate (Ti/Fe = 15)	6	240	1.70	1.93	0.587	[87]
Prussian-blue/hydroxyapatite/alginate *	SW	5	0.308	0.334	1.31	[28]
Crown ether pillared Zr phosphonate	0.2 M HNO_3_	120	1.58	1.56	3.79	[53]
ALG-PEI	7	90	0.992	1.31	0.430	This work
APO-PEI	7	90	2.39	2.55	2.05	This work

n.d.: not documented; *: seawater sample spiked with both Cs(I) and Sr(II); time: min; q_m_: mmol Cs g^−1^; b_L_: L mmol^−1^.

### Appendix B.4. Sorption Selectivity

**Table A7:** Summary of main conclusions on selectivity issues.

pH	pH_eq_ ≈ 3.2–3.3			pH_eq_ ≈ 8.2–8.3		
Sorbent	Functional Groups	Mechanism	Affinity Sensitivity	Functional Groups	Mechanism	Affinity Sensitivity
ALG-PEI	R-COOH, R-NH_3_^+^, R-NH_2_^±^, R_2_NH^±^ R-OH	IE	Na(I) Fe(III) Ca(II) Al(III)	R-COO^−^, R-NH_2_, R-NH^±^, R_2_NH^±^ R-OH	IE, C	Fe(III) Al(III) Na(I) Ca(II)
APO-PEI	R-COOH, R-NH_3_^+^, R-NH_2_^±^, R_2_NH^±^ R-OH, R-PO_3_H^−^	IE, C	Sr(II) Cs(I) Na(I) Fe(III) Al(III)	R-COO^−^, R-NH_2_, RNH^±^, R_2_NH^±^ R-OH, R-PO_3_^2−^	IE, C	Sr(II) Cs(I) Na(I) Al(III) Fe(III)

Mechanisms: IE, Ion-exchange; C, Chelation.

## Appendix C. Application to Seawater Sample

**Table A8:** Composition of the seawater sample.

Element	Na	K	Mg	Ca	B	U	As	Sr	Cs
Conc. (mg L^−1^)	11530	689	1294	452.7	3.022	0.0091	0.0776	4.326	0.326
Conc. (mmol L^−1^)	501.5	22.89	53.22	11.30	0.2796	0.0382 *	10.4 *	49.4 *	2.45 *
MR	2.04 × 10^5^	9333	21,697	4605	114	0.0156	0.422	20.1	1

*: µmol L^−1^ unit; MR: molar ratio (element/Cs).
